# Supplementary material for: What does it take to learn the rules of RNA base pairing? A lot less than you may think
Source: bioRxiv. 2025 Aug 2:2025.07.31.668042. Preprint. [Version 1] doi: 10.1101/2025.07.31.668042 (PMC12324431; doi:10.1101/2025.07.31.668042)

WARNING: R2R is not intended to evaluate evidence for covariation or RNA structure where this is in question. It is not appropriate to use R2R´s covariation markings to declare that there is evidence of structural conservation within an alignment.
R2R is a drawing program. As the original paper, Weinberg and Breaker, 2011 wrote:
"This automated R2R annotation [of covariation] does not reflect the extent or confidence of covariation. While such information can be useful, we believe that thorough evaluation of covariation evidence ultimately requires analysis of the full sequence alignment. For example, misleading covariation can result from an incorrect alignment of sequences, or from alignments of sequences that do not function as structured RNAs. Unfortunately, there is no accepted method to assign confidence that entirely eliminates the need to analyze the full alignment."
To disable this warning, run r2r with --disable-usage-warning

SAM-IV NC_003888.3/2308784-2308334 multistem=original

Shrinking nucs & bonds using
nucShrinkWithCircleNuc and pairBondScaleWithOneSeq
because circle_nuc or #=GR ... CLEAVAGE was used
To disable this warning message:
#=GF R2R SetDrawingParam nucShrinkWithCircleNuc 1
See note1 in manual for more information.

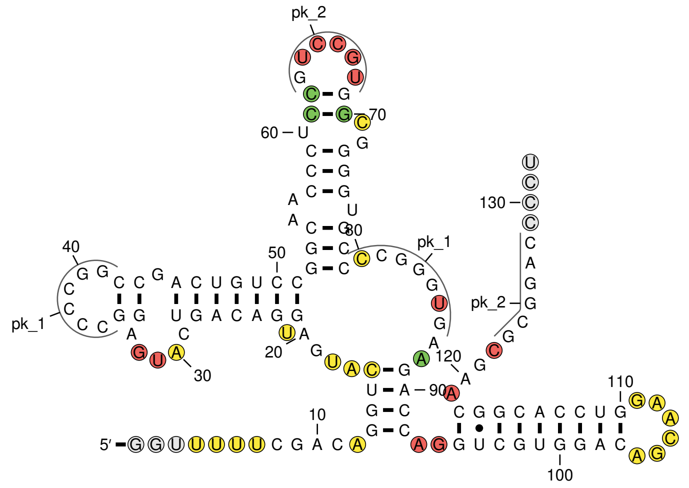

SAM-IV NC_003888.3/2308784-2308334 multistem=solver internalloop=solver2

Shrinking nucs & bonds using
nucShrinkWithCircleNuc and pairBondScaleWithOneSeq
because circle_nuc or #=GR ... CLEAVAGE was used
To disable this warning message:
#=GF R2R SetDrawingParam nucShrinkWithCircleNuc 1 drawingParams.pairBondScaleWithCircleNuc 1
See note1 in manual for more information.

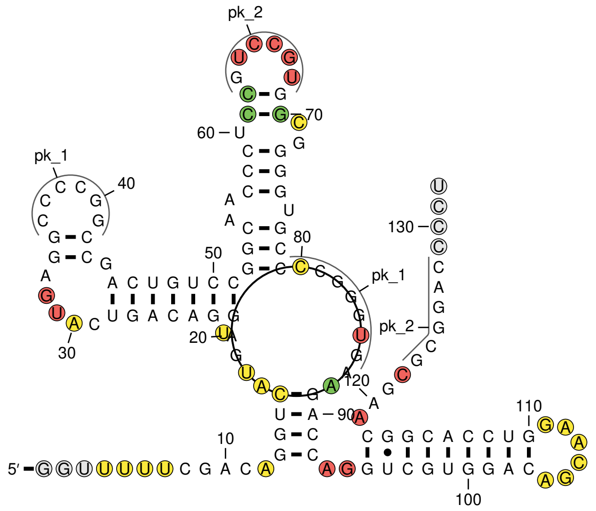

SAM-IV NC_003888.3/2308784-2308334 pseudoknot=1

Shrinking nucs & bonds using
nucShrinkWithCircleNuc and pairBondScaleWithOneSeq
because circle_nuc or #=GR ... CLEAVAGE was used
To disable this warning message:
#=GF R2R SetDrawingParam nucShrinkWithCircleNuc 1 drawingParams.pairBondScaleWithCircleNuc 1
See note1 in manual for more information.

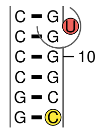

SAM-IV NC_003888.3/2308784-2308334 pseudoknot=2

Shrinking nucs & bonds using
nucShrinkWithCircleNuc and pairBondScaleWithOneSeq
because circle_nuc or #=GR ... CLEAVAGE was used
To disable this warning message:
#=GF R2R SetDrawingParam nucShrinkWithCircleNuc 1 drawingParams.pairBondScaleWithCircleNuc 1
See note1 in manual for more information.

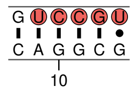

SAM-IV.cons

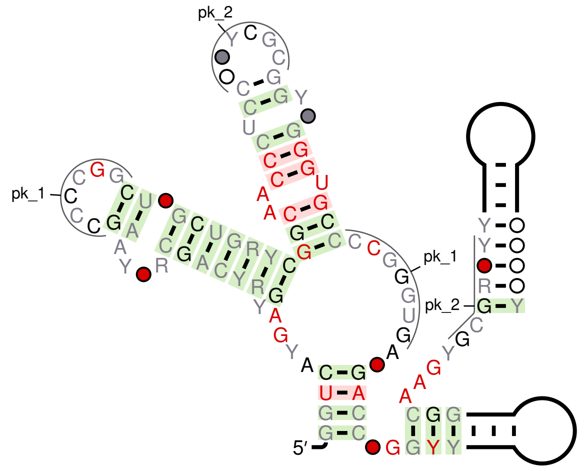

SAM-IV.cons NC_003888.3/2308784-2308334

Shrinking nucs & bonds using
nucShrinkWithCircleNuc and pairBondScaleWithOneSeq
because circle_nuc or #=GR ... CLEAVAGE was used
To disable this warning message:
#=GF R2R SetDrawingParam nucShrinkWithCircleNuc 1 drawingParams.pairBondScaleWithCircleNuc 1
See note1 in manual for more information.

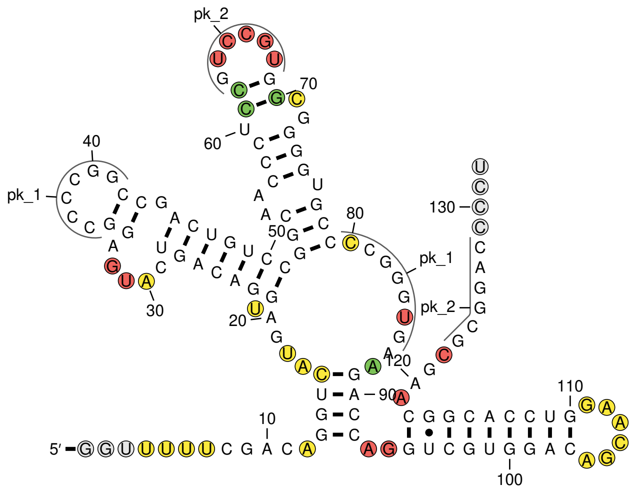

SAM-IV.cons Weinberg2008-Fig1=1 multistem=original

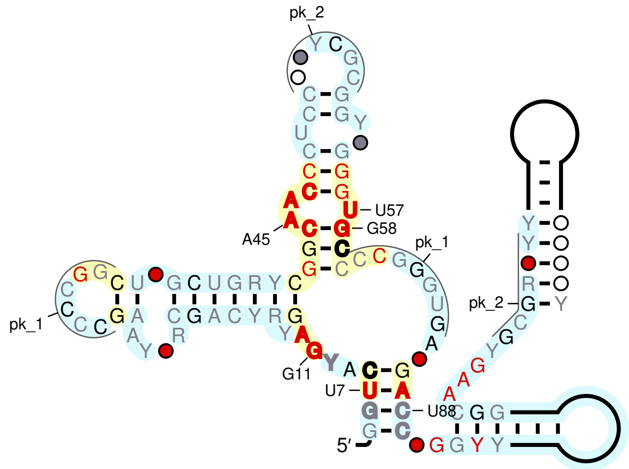

SAM-IV.cons multistem=bulgey

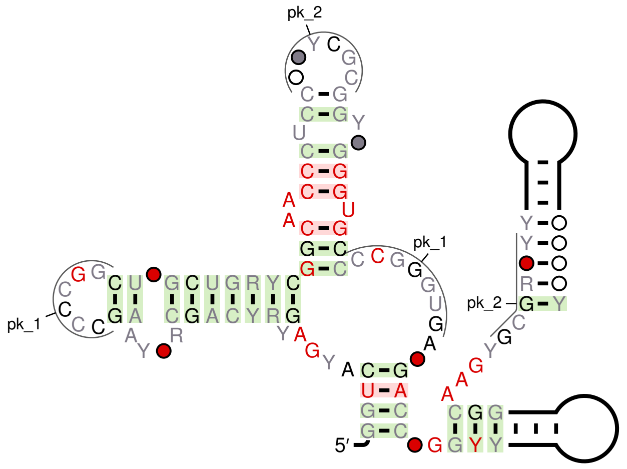

SAM-IV.cons multistem=original

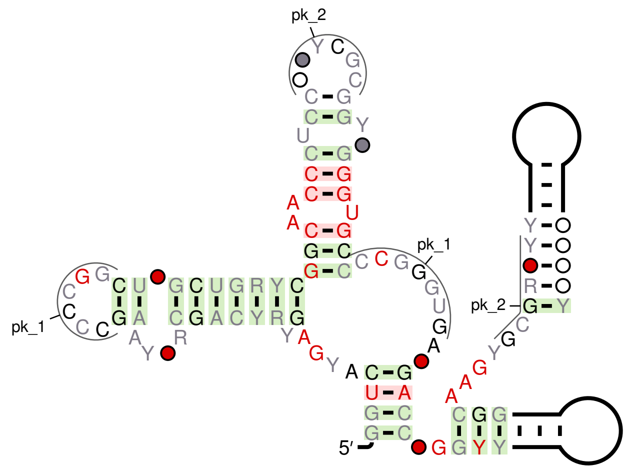

SAM-IV.cons multistem=original internalloop=solver

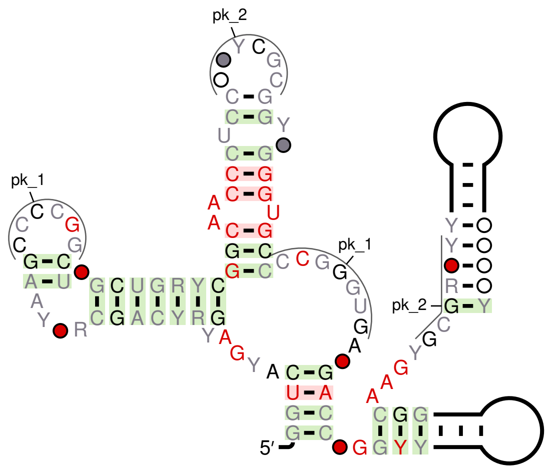

SAM-IV.cons multistem=original internalloop=solver2

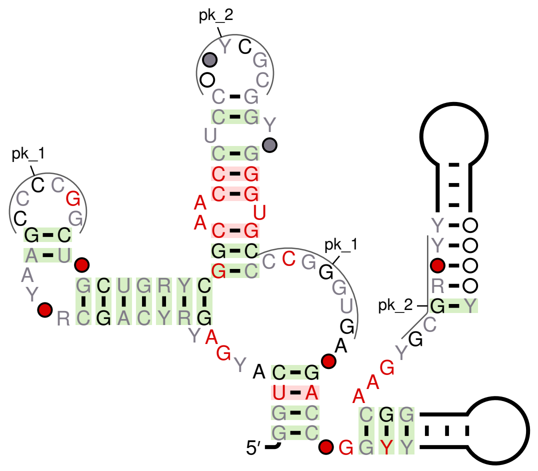

SAM-IV.cons multistem=original internalloop=solver3

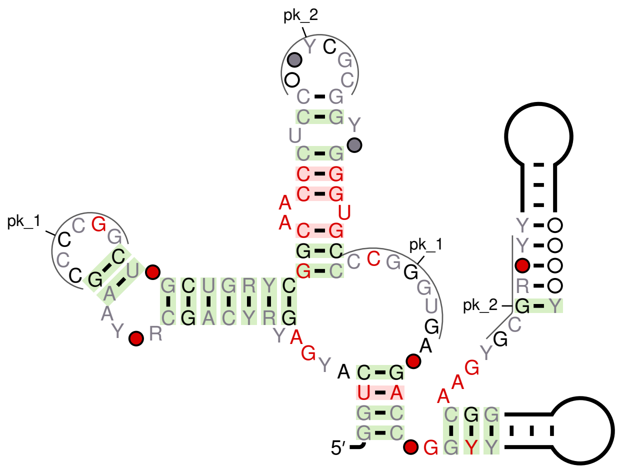

SAM-IV.cons multistem=original internalloop=turn

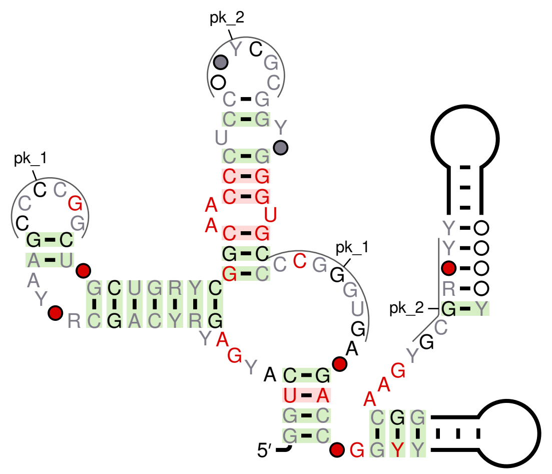

SAM-IV.cons multistem=solver

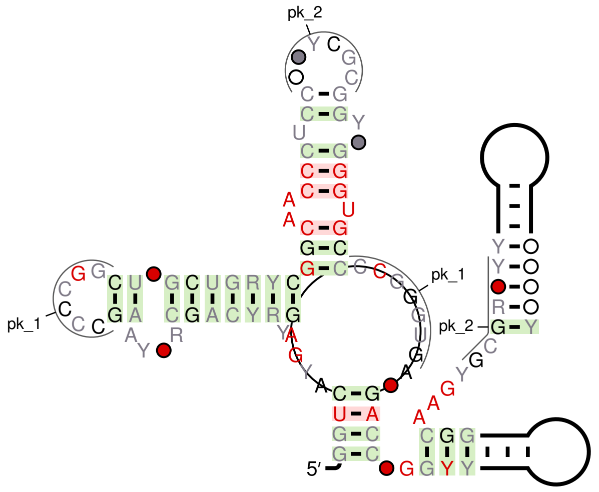

SAM-IV.cons multistem=solver2

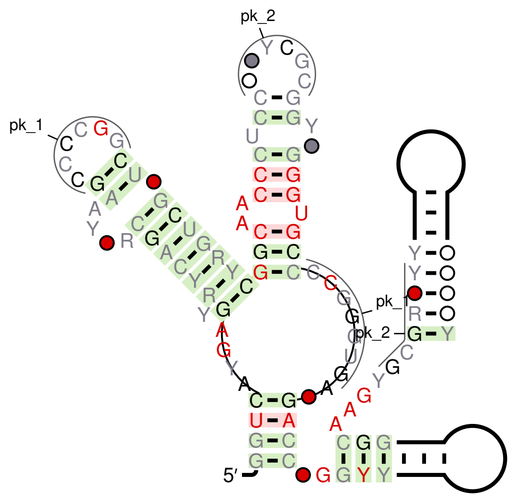

SAM-IV.cons pseudoknot=1

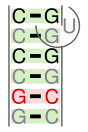

SAM-IV.cons pseudoknot=2

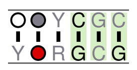

SAM-IV.cons skeleton-with-bp

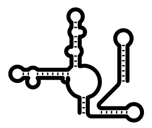

WARNING: R2R is not intended to evaluate evidence for covariation or RNA structure where this is in question. It is not appropriate to use R2R´s covariation markings to declare that there is evidence of structural conservation within an alignment. R2R is a drawing program. As the original paper, Weinberg and Breaker, 2011 wrote:
"This automated R2R annotation [of covariation] does not reflect the extent or confidence of covariation. While such information can be useful, we believe that thorough evaluation of covariation evidence ultimately requires analysis of the full sequence alignment. For example, misleading covariation can result from an incorrect alignment of sequences, or from alignments of sequences that do not function as structured RNAs. Unfortunately, there is no accepted method to assign confidence that entirely eliminates the need to analyze the full alignment."
To disable this warning, run r2r with --disable-usage-warning

HEARO-RYGCAA.cons

subfam_weight=0.0745961

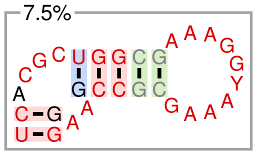

HEARO-UAA.cons

subfam_weight=0.732286

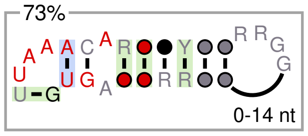

HEARO.cons

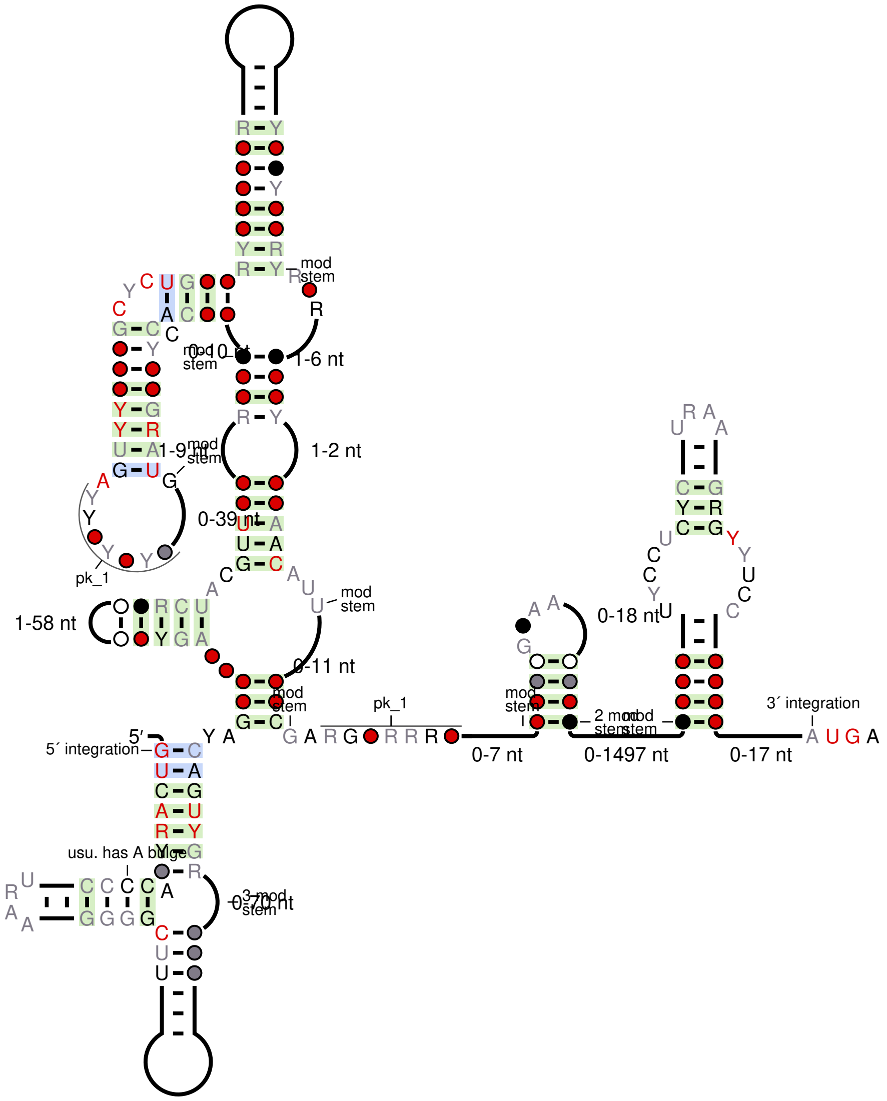

HEARO.cons NZ_ABYK01000021.1/61949-61536

Shrinking nucs & bonds using
nucShrinkWithCircleNuc and pairBondScaleWithOneSeq
because circle_nuc or #=GR ... CLEAVAGE was used
To disable this warning message:
#=GF R2R SetDrawingParam nucShrinkWithCircleNuc 1 drawingParams.pairBondScaleWithCircleNuc 1
See note1 in manual for more information.

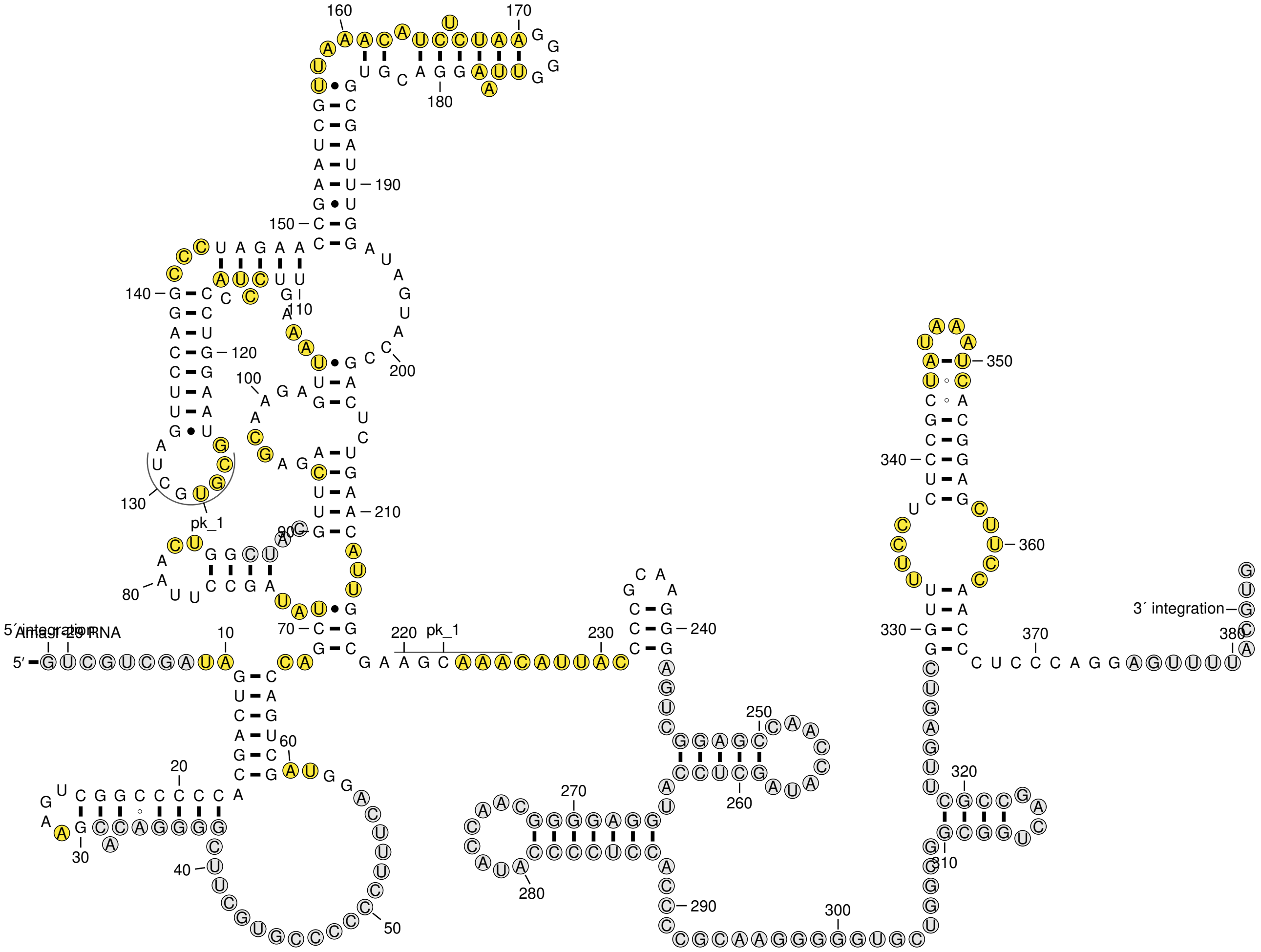

HEARO.cons NZ_ABYK01000021.1/61949-61536 pknot=1

Shrinking nucs & bonds using
nucShrinkWithCircleNuc and pairBondScaleWithOneSeq
because circle_nuc or #=GR ... CLEAVAGE was used
To disable this warning message:
#=GF R2R SetDrawingParam nucShrinkWithCircleNuc 1 drawingParams.pairBondScaleWithCircleNuc 1
See note1 in manual for more information.

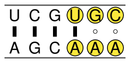

HEARO.cons pknot=1

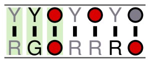

HEARO.cons skeleton-with-bp

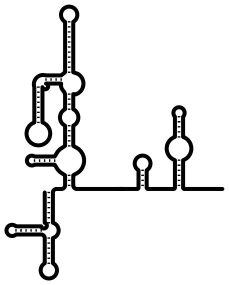

WARNING: R2R is not intended to evaluate evidence for covariation or RNA structure where this is in question. It is not appropriate to use R2R's covariation markings to declare that there is evidence of structural conservation within an alignment. R2R is a drawing program. As the original paper, Weinberg and Breaker, 2011 wrote:
"This automated R2R annotation [of covariation] does not reflect the extent or confidence of covariation. While such information can be useful, we believe that thorough evaluation of covariation evidence ultimately requires analysis of the full sequence alignment. For example, misleading covariation can result from an incorrect alignment of sequences, or from alignments of sequences that do not function as structured RNAs. Unfortunately, there is no accepted method to assign confidence that entirely eliminates the need to analyze the full alignment."
To disable this warning, run r2r with --disable-usage-warning

GOLLD-3vnottruncated.cons
subfam_weight=0.689503
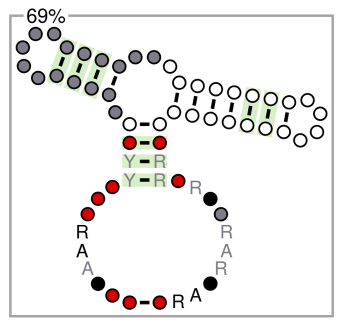

GOLLD-3vp1.cons
subfam_weight=0.118998
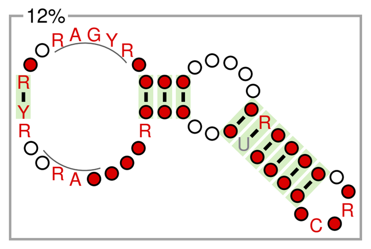

GOLLD-3vp12.cons
subfam_weight=0.0697332
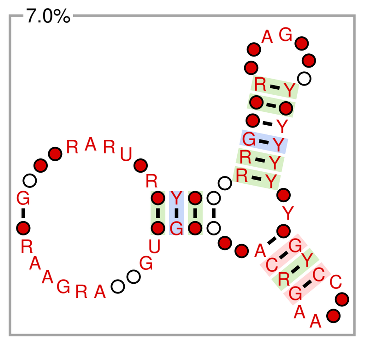

GOLLD-3vp23.cons
subfam_weight=0.401602
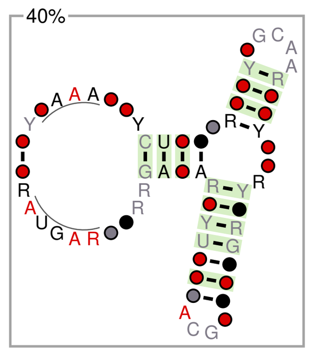

GOLLD-d3classic.cons
subfam_weight=0.192677
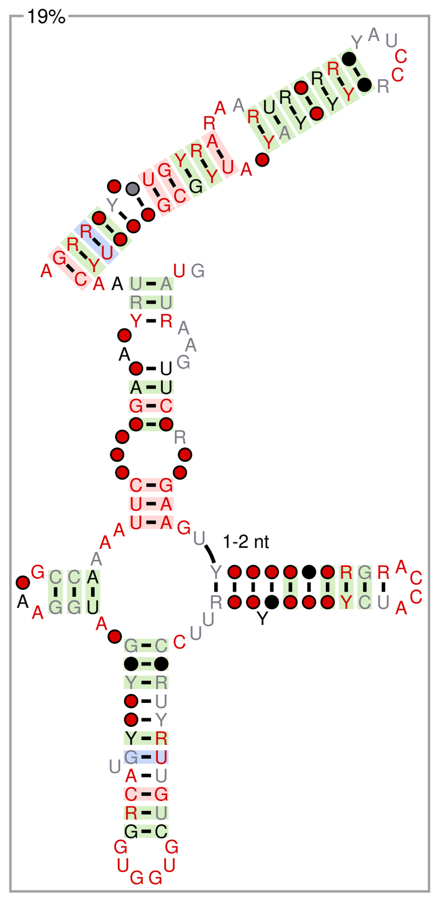

GOLLD-d3justone.cons
subfam_weight=0.133952
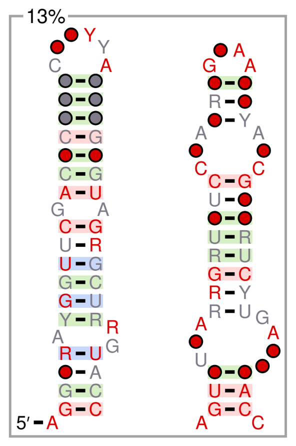

GOLLD-d3valid.cons
subfam_weight=0.326629
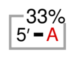

GOLLD-p-1-surround.cons
subfam_weight=0.509432
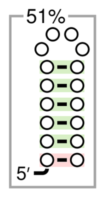

GOLLD-p-1.cons
subfam_weight=0.452781
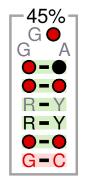

GOLLD-p-2.cons
subfam_weight=0.133952
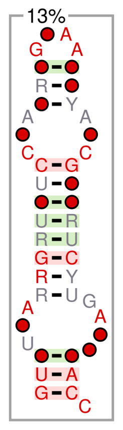

GOLLD-pknot1.cons
subfam_weight=1
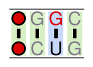

GOLLD-pknot2.cons
subfam_weight=1
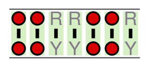

GOLLD-pknot3.cons
subfam_weight=1
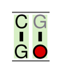

GOLLD-pknot4.cons
subfam_weight=1
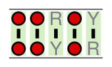

GOLLD-pknot5.cons
subfam_weight=1
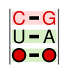

GOLLD-skipbadd2.cons
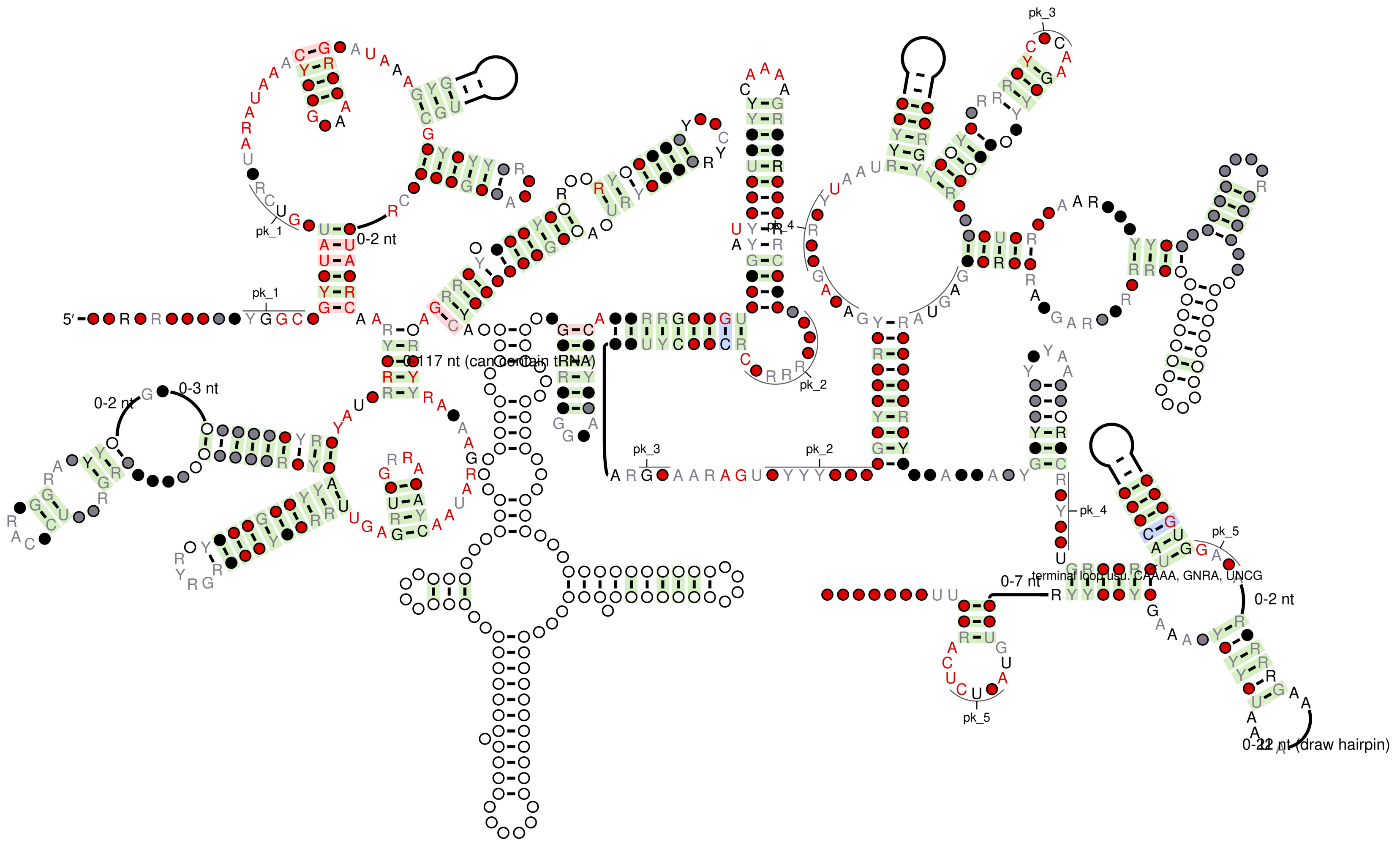

GOLLD.cons
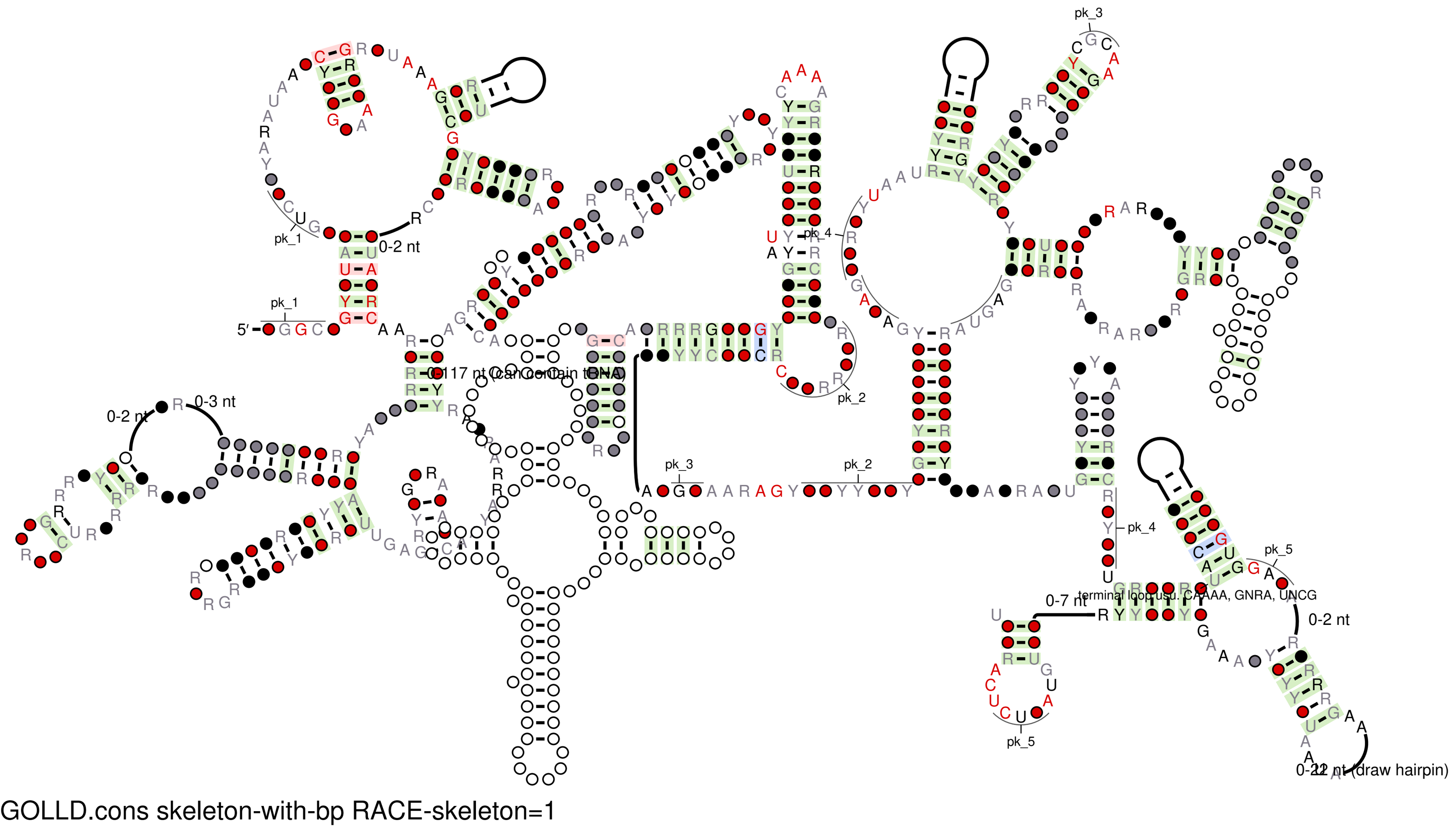

GOLLD.cons NC_008497.1/1119234-1118463:3
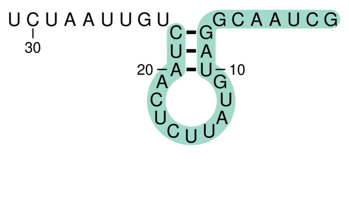

GOLLD.cons NC_008497.1/1119234-1118463:5
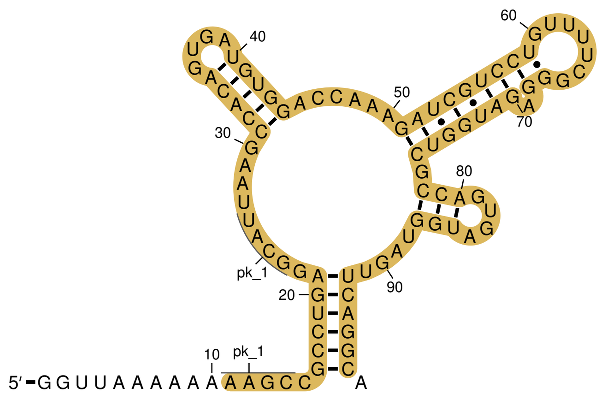

GOLLD.cons skeleton-with-bp
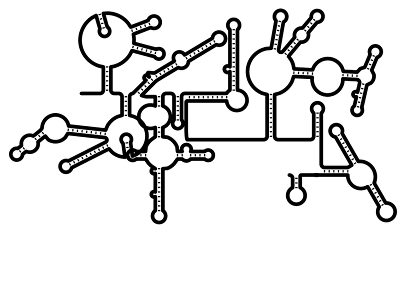

GOLLD.cons skeleton-with-bp RACE-skeleton=1
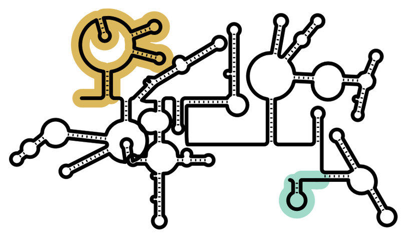

GOLLD.cons skeleton-with-bp align-skeleton=1
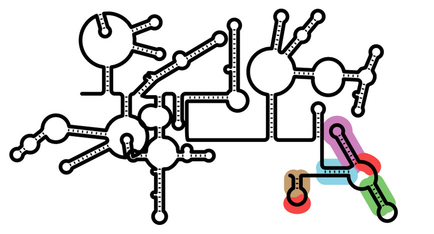

WARNING: R2R is not intended to evaluate evidence for covariation or RNA structure where this is in question. It is not appropriate to use R2R´s covariation markings to declare that there is evidence of structural conservation within an alignment. R2R is a drawing program. As the original paper, Weinberg and Breaker, 2011 wrote:
"This automated R2R annotation [of covariation] does not reflect the extent or confidence of covariation. While such information can be useful, we believe that thorough evaluation of covariation evidence ultimately requires analysis of the full sequence alignment. For example, misleading covariation can result from an incorrect alignment of sequences, or from alignments of sequences that do not function as structured RNAs. Unfortunately, there is no accepted method to assign confidence that entirely eliminates the need to analyze the full alignment."
To disable this warning, run r2r with
--disable-usage-warning

IMES-1-pknot.cons
subfam_weight=1

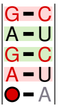

IMES-1.cons

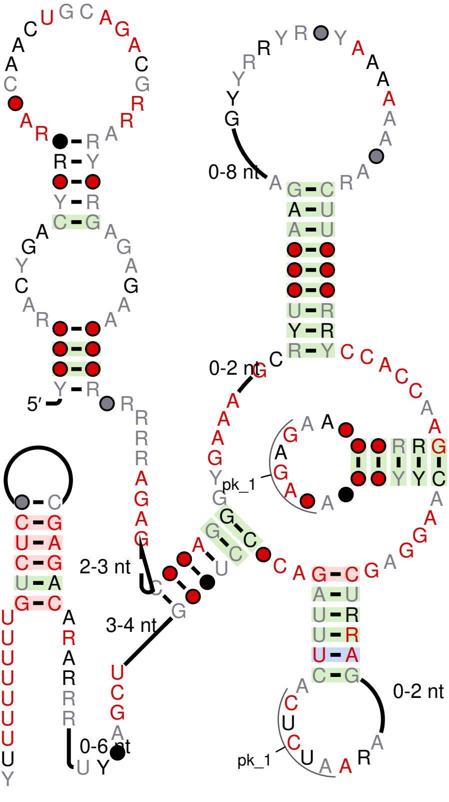

WARNING: R2R is not intended to evaluate evidence for covariation or RNA structure where this is in question. It is not appropriate to use R2R´s covariation markings to declare that there is evidence of structural conservation within an alignment. R2R is a drawing program. As the original paper, Weinberg and Breaker, 2011 wrote:
"This automated R2R annotation [of covariation] does not reflect the extent or confidence of covariation. While such information can be useful, we believe that thorough evaluation of covariation evidence ultimately requires analysis of the full sequence alignment. For example, misleading covariation can result from an incorrect alignment of sequences, or from alignments of sequences that do not function as structured RNAs. Unfortunately, there is no accepted method to assign confidence that entirely eliminates the need to analyze the full alignment."
To disable this warning, run r2r with
--disable-usage-warning

Moco-withP3.cons
subfam_weight=0.496344

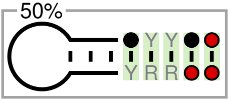

Moco.cons

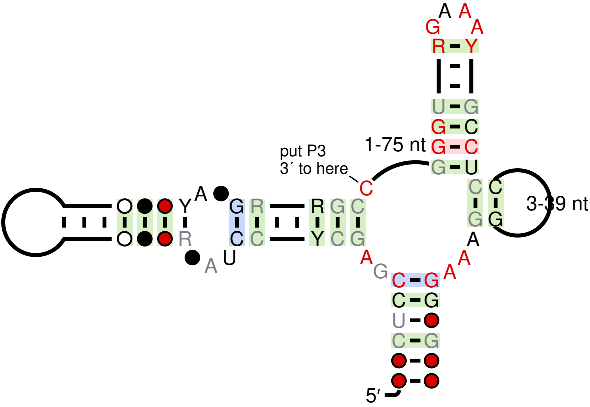

WARNING: R2R is not intended to evaluate evidence for covariation or RNA structure where this is in question. It is not appropriate to use R2R´s covariation markings to declare that there is evidence of structural conservation within an alignment. R2R is a drawing program. As the original paper, Weinberg and Breaker, 2011 wrote:

"This automated R2R annotation [of covariation] does not reflect the extent or confidence of covariation. While such information can be useful, we believe that thorough evaluation of covariation evidence ultimately requires analysis of the full sequence alignment. For example, misleading covariation can result from an incorrect alignment of sequences, or from alignments of sequences that do not function as structured RNAs. Unfortunately, there is no accepted method to assign confidence that entirely eliminates the need to analyze the full alignment."

To disable this warning, run r2r with --disable-usage-warning

demo-multistem-HEARO2.cons

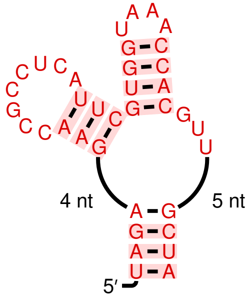

demo-multistem-HEARO2.cons solver=1

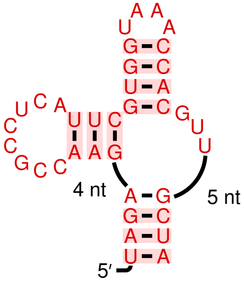

demo-multistem-HEARO2.cons solver=2

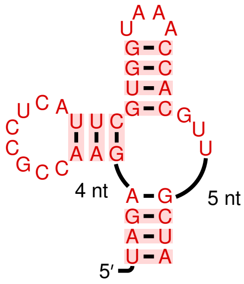

WARNING: R2R is not intended to evaluate evidence for covariation or RNA structure where this is in question. It is not appropriate to use R2R´s covariation markings to declare that there is evidence of structural conservation within an alignment. R2R is a drawing program. As the original paper, Weinberg and Breaker, 2011 wrote:
"This automated R2R annotation [of covariation] does not reflect the extent or confidence of covariation. While such information can be useful, we believe that thorough evaluation of covariation evidence ultimately requires analysis of the full sequence alignment. For example, misleading covariation can result from an incorrect alignment of sequences, or from alignments of sequences that do not function as structured RNAs. Unfortunately, there is no accepted method to assign confidence that entirely eliminates the need to analyze the full alignment."
To disable this warning, run r2r with --disable-usage-warning

yjdF-AUG.cons
subfam_weight=0.832593

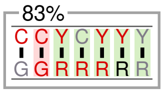

yjdF-altstem.cons
subfam_weight=1

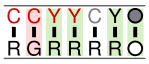

yjdF.cons

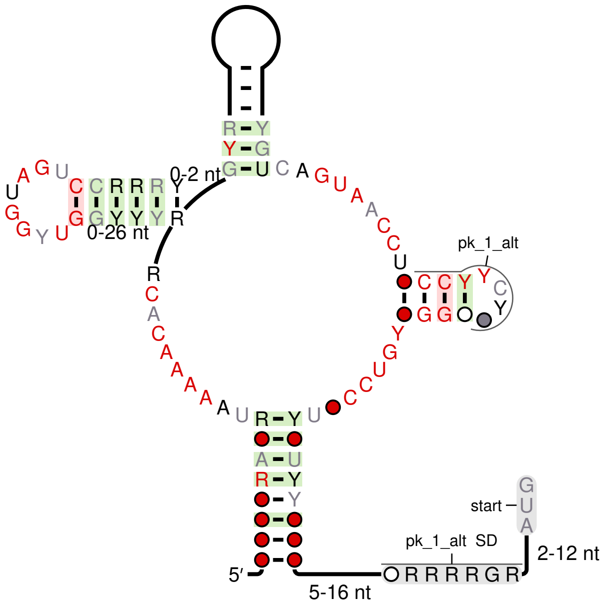

yjdF.cons NC_000964.2/1274965-1275471
Shrinking nucs & bonds using
nucShrinkWithCircleNuc and pairBondScaleWithOneSeq
because circle_nuc or #=GR ... CLEAVAGE was used
To disable this warning message:
#=GF R2R SetDrawingParam nucShrinkWithCircleNuc 1 drawingParams.pairBondScaleWithCircleNuc 1
See note1 in manual for more information.

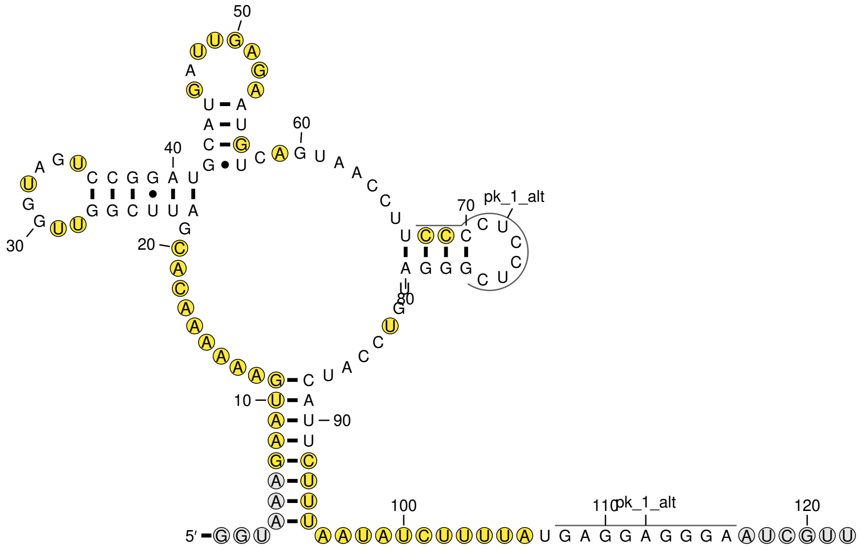

yjdF.cons NC_000964.2/1274965-1275471:L
Shrinking nucs & bonds using
nucShrinkWithCircleNuc and pairBondScaleWithOneSeq
because circle_nuc or #=GR ... CLEAVAGE was used
To disable this warning message:
#=GF R2R SetDrawingParam nucShrinkWithCircleNuc 1 drawingParams.pairBondScaleWithCircleNuc 1
See note1 in manual for more information.

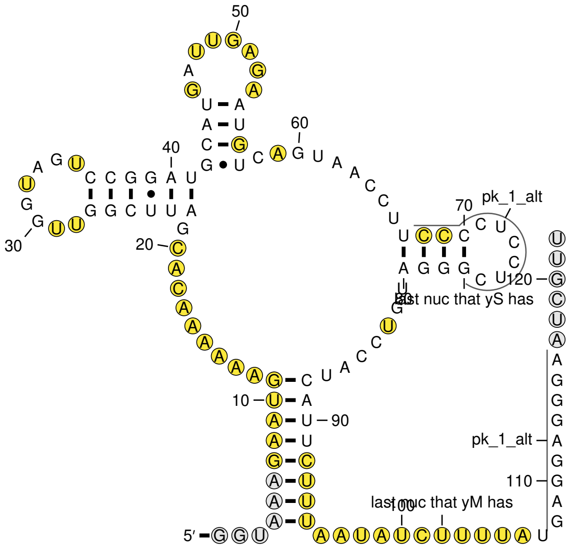

yjdF.cons NC_000964.2/1274965-1275471:M
Shrinking nucs & bonds using
nucShrinkWithCircleNuc and pairBondScaleWithOneSeq
because circle_nuc or #=GR ... CLEAVAGE was used
To disable this warning message:
#=GF R2R SetDrawingParam nucShrinkWithCircleNuc 1 drawingParams.pairBondScaleWithCircleNuc 1
See note1 in manual for more information.

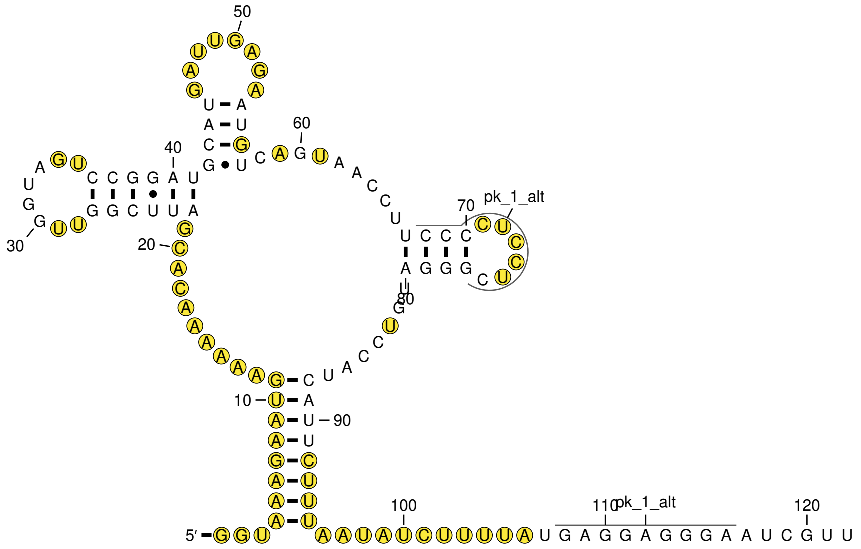

yjdF.cons NC_000964.2/1274965-1275471:S

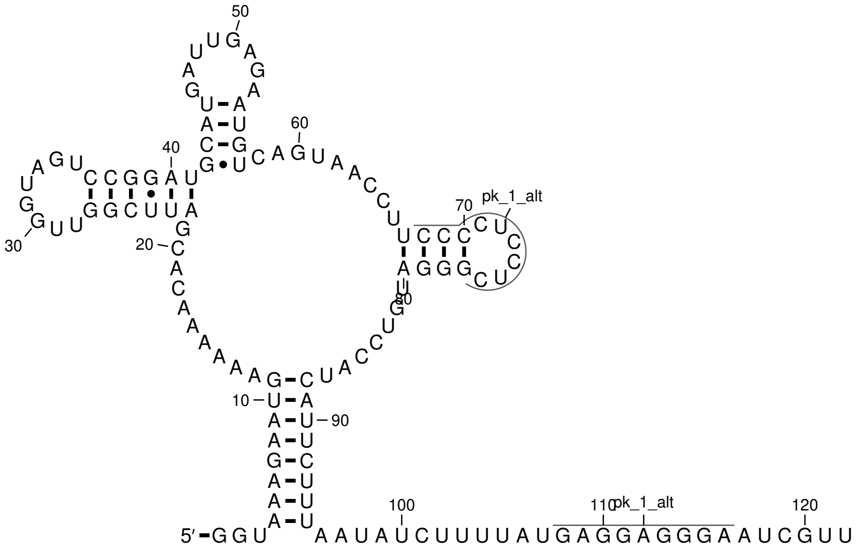

WARNING: R2R is not intended to evaluate evidence for covariation or RNA structure where this is in question. It is not appropriate to use R2R´s covariation markings to declare that there is evidence of structural conservation within an alignment. R2R is a drawing program. As the original paper, Weinberg and Breaker, 2011 wrote:
"This automated R2R annotation [of covariation] does not reflect the extent or confidence of covariation. While such information can be useful, we believe that thorough evaluation of covariation evidence ultimately requires analysis of the full sequence alignment. For example, misleading covariation can result from an incorrect alignment of sequences, or from alignments of sequences that do not function as structured RNAs. Unfortunately, there is no accepted method to assign confidence that entirely eliminates the need to analyze the full alignment."
To disable this warning, run r2r with
--disable-usage-warning

Dictyoglomi-1.cons

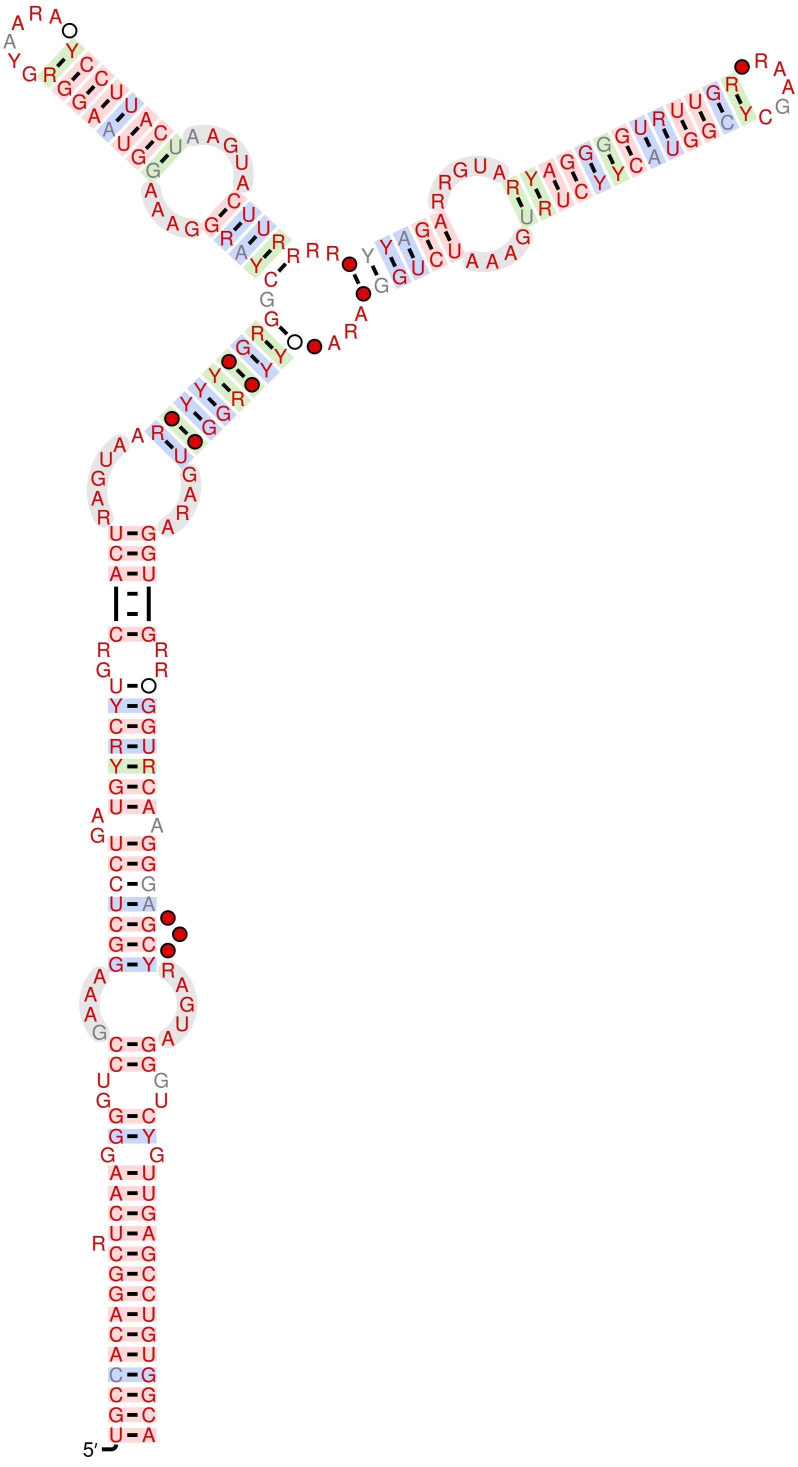

WARNING: R2R is not intended to evaluate evidence for covariation or RNA structure where this is in question. It is not appropriate to use R2R's covariation markings to declare that there is evidence of structural conservation within an alignment. R2R is a drawing program. As the original paper, Weinberg and Breaker, 2011 wrote:
"This automated R2R annotation [of covariation] does not reflect the extent or confidence of covariation. While such information can be useful, we believe that thorough evaluation of covariation evidence ultimately requires analysis of the full sequence alignment. For example, misleading covariation can result from an incorrect alignment of sequences, or from alignments of sequences that do not function as structured RNAs. Unfortunately, there is no accepted method to assign confidence that entirely eliminates the need to analyze the full alignment."
To disable this warning, run r2r with --disable-usage-warning

manA-pknot1.cons
subfam_weight=0.891987

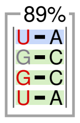

manA-pknot2.cons
subfam_weight=1

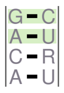

manA-var1.cons
subfam_weight=0.028518

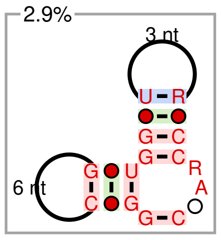

manA-var2.cons
subfam_weight=0.891987

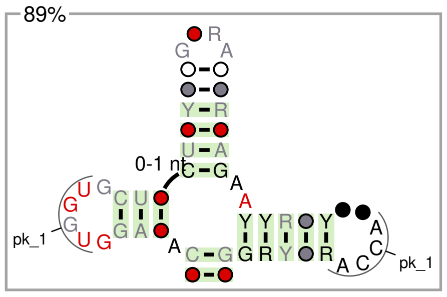

manA-var3.cons
subfam_weight=0.0794949

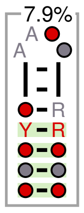

manA.cons

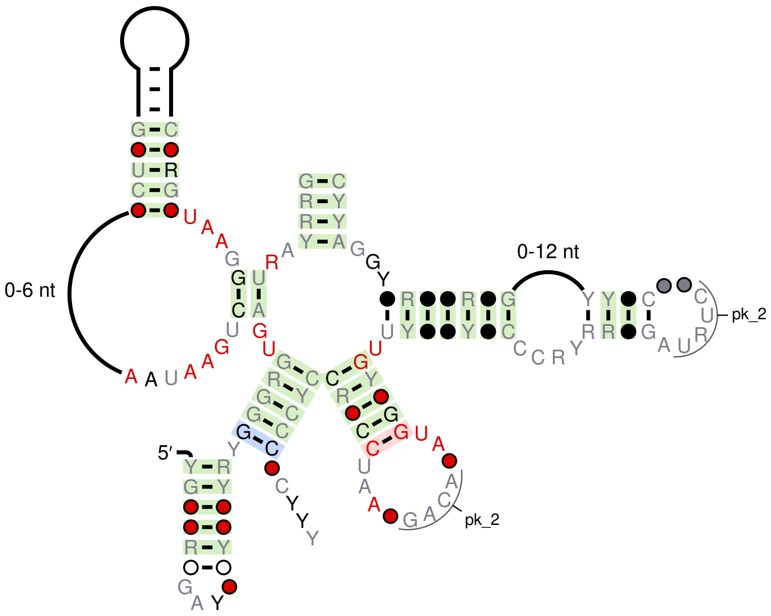

manA.cons NZ_AAOJ01000001.1/468755-469351

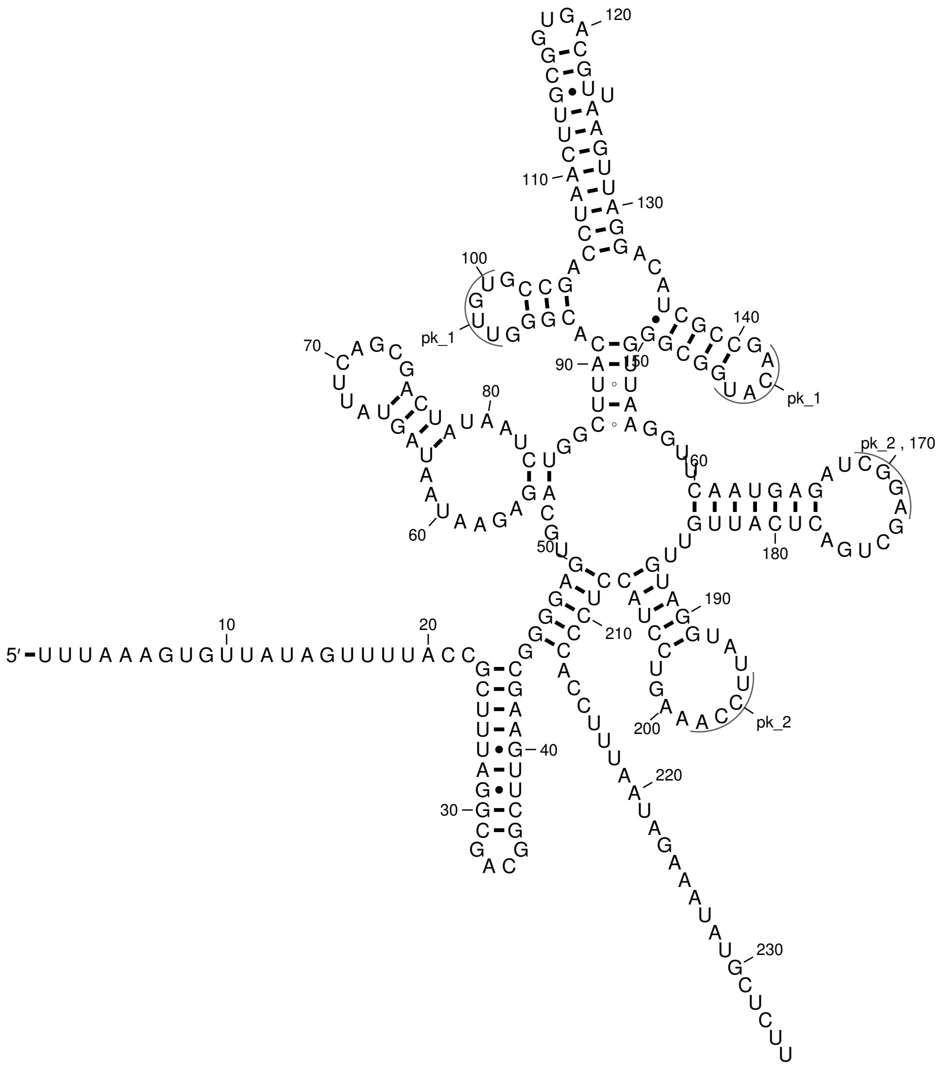

manA.cons skeleton varhairpin=1 var-right-stem=1

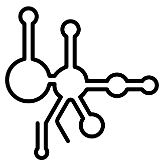

manA.cons skeleton-with-bp varhairpin=1 var-right-stem=1

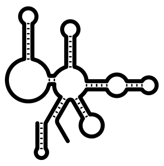

manA.cons varhairpin=1 var-right-stem=1

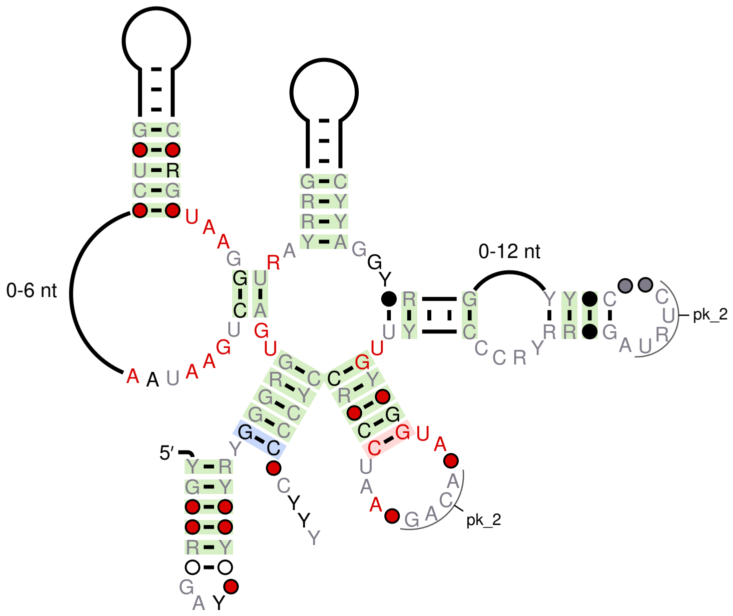

WARNING: R2R is not intended to evaluate evidence for covariation or RNA structure where this is in question. It is not appropriate to use R2R´s covariation markings to declare that there is evidence of structural conservation within an alignment. R2R is a drawing program. As the original paper, Weinberg and Breaker, 2011 wrote:
"This automated R2R annotation [of covariation] does not reflect the extent or confidence of covariation. While such information can be useful, we believe that thorough evaluation of covariation evidence ultimately requires analysis of the full sequence alignment. For example, misleading covariation can result from an incorrect alignment of sequences, or from alignments of sequences that do not function as structured RNAs. Unfortunately, there is no accepted method to assign confidence that entirely eliminates the need to analyze the full alignment."
To disable this warning, run r2r with
--disable-usage-warning

traJ-II.cons

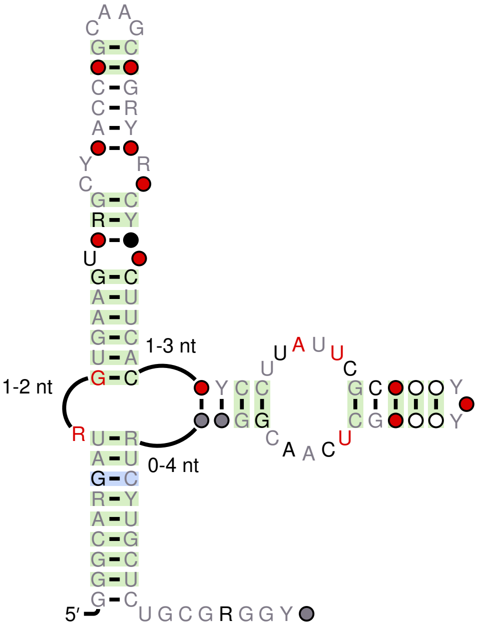

WARNING: R2R is not intended to evaluate evidence for covariation or RNA structure where this is in question. It is not appropriate to use R2R´s covariation markings to declare that there is evidence of structural conservation within an alignment. R2R is a drawing program. As the original paper, Weinberg and Breaker, 2011 wrote:
"This automated R2R annotation [of covariation] does not reflect the extent or confidence of covariation. While such information can be useful, we believe that thorough evaluation of covariation evidence ultimately requires analysis of the full sequence alignment. For example, misleading covariation can result from an incorrect alignment of sequences, or from alignments of sequences that do not function as structured RNAs. Unfortunately, there is no accepted method to assign confidence that entirely eliminates the need to analyze the full alignment."
To disable this warning, run r2r with --disable-usage-warning

Bacteroidales-1-pknot.cons
subfam_weight=1

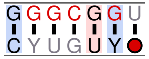

Bacteroidales-1.cons

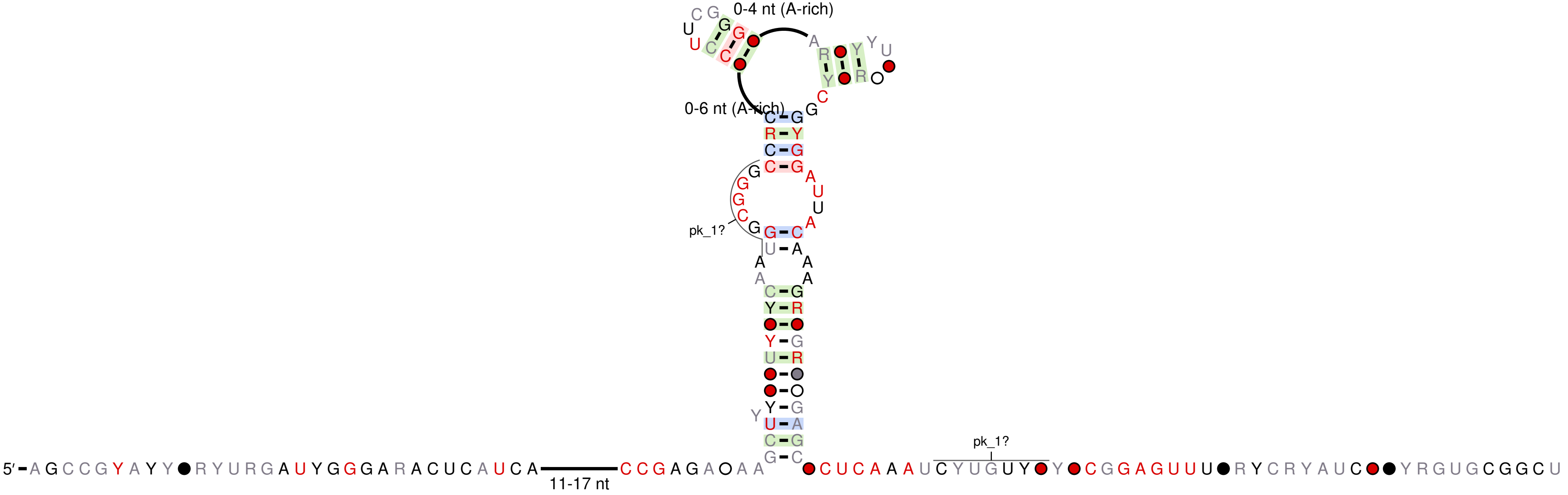

WARNING: R2R is not intended to evaluate evidence for covariation or RNA structure where this is in question. It is not appropriate to use R2R´s covariation markings to declare that there is evidence of structural conservation within an alignment. R2R is a drawing program. As the original paper, Weinberg and Breaker, 2011 wrote:
"This automated R2R annotation [of covariation] does not reflect the extent or confidence of covariation. While such information can be useful, we believe that thorough evaluation of covariation evidence ultimately requires analysis of the full sequence alignment. For example, misleading covariation can result from an incorrect alignment of sequences, or from alignments of sequences that do not function as structured RNAs. Unfortunately, there is no accepted method to assign confidence that entirely eliminates the need to analyze the full alignment."
To disable this warning, run r2r with
--disable-usage-warning

potC.cons

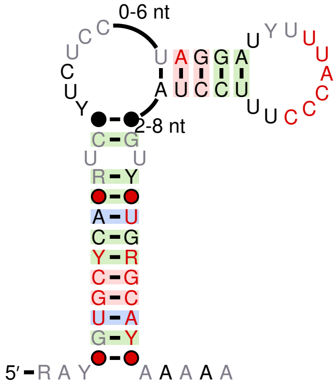

WARNING: R2R is not intended to evaluate evidence for covariation or RNA structure where this is in question. It is not appropriate to use R2R´s covariation markings to declare that there is evidence of structural conservation within an alignment. R2R is a drawing program. As the original paper, Weinberg and Breaker, 2011 wrote:
"This automated R2R annotation [of covariation] does not reflect the extent or confidence of covariation. While such information can be useful, we believe that thorough evaluation of covariation evidence ultimately requires analysis of the full sequence alignment. For example, misleading covariation can result from an incorrect alignment of sequences, or from alignments of sequences that do not function as structured RNAs. Unfortunately, there is no accepted method to assign confidence that entirely eliminates the need to analyze the full alignment."
To disable this warning, run r2r with
--disable-usage-warning

epsC-pknot.cons

subfam_weight=1

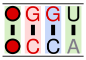

epsC.cons

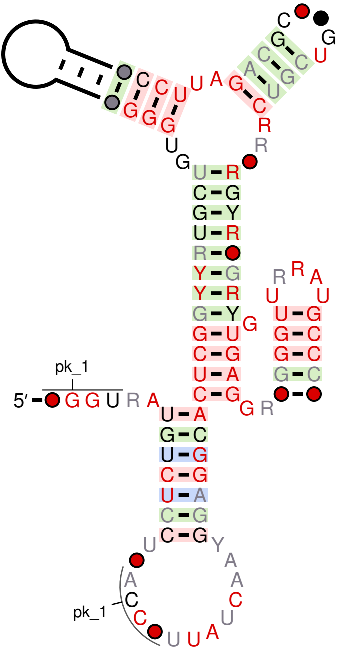

WARNING: R2R is not intended to evaluate evidence for covariation or RNA structure where this is in question. It is not appropriate to use R2R´s covariation markings to declare that there is evidence of structural conservation within an alignment. R2R is a drawing program. As the original paper, Weinberg and Breaker, 2011 wrote:
"This automated R2R annotation [of covariation] does not reflect the extent or confidence of covariation. While such information can be useful, we believe that thorough evaluation of covariation evidence ultimately requires analysis of the full sequence alignment. For example, misleading covariation can result from an incorrect alignment of sequences, or from alignments of sequences that do not function as structured RNAs. Unfortunately, there is no accepted method to assign confidence that entirely eliminates the need to analyze the full alignment."
To disable this warning, run r2r with --disable-usage-warning

crcB NC_008578.1/805741-806202 alt-struct=2

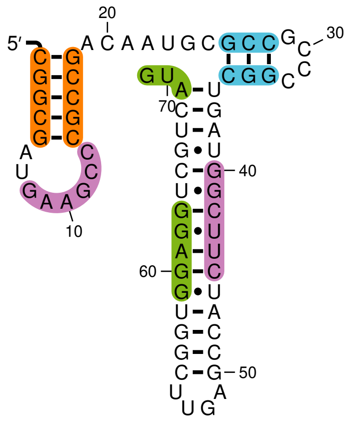

crcB NZ_ABYJ01000311.1/4968-4495 alt-struct=2

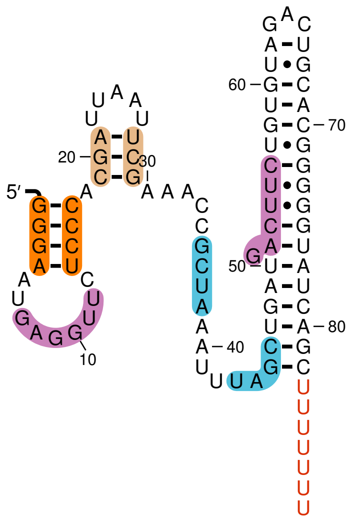

crcB NZ_ABYJ01000311.1/4968-4495 no-shading=1

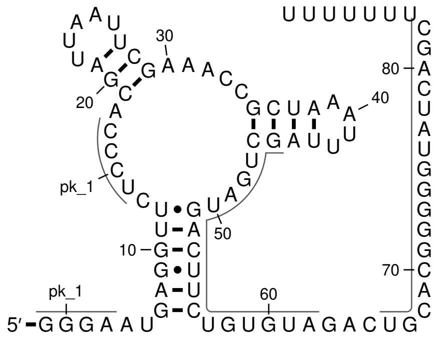

crcB NZ_ABYJ01000311.1/4968-4495 no-shading=1 anyangle=1

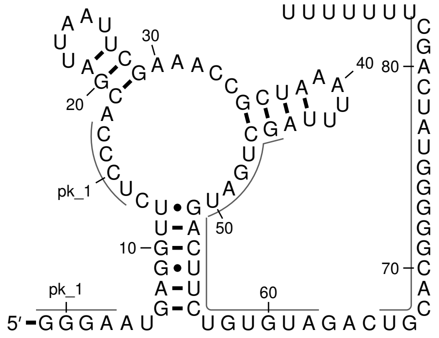

crcB NZ_ABYJ01000311.1/4968-4495 no-shading=1 anyangle=1 drawcirc=1

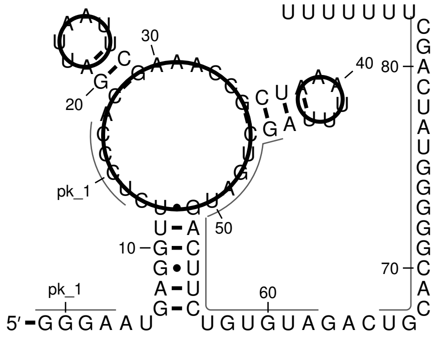

crcB NZ_ABYJ01000311.1/4968-4495 no-shading=1 anyangle=1 drawedges=1

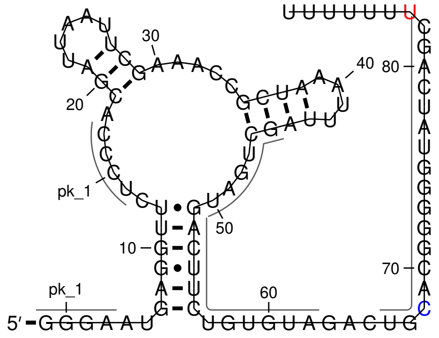

crcB-P2.cons
subfam_weight=0.308697

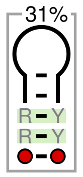

crcB-pknot1.cons
subfam_weight=1

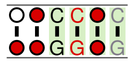

crcB.cons

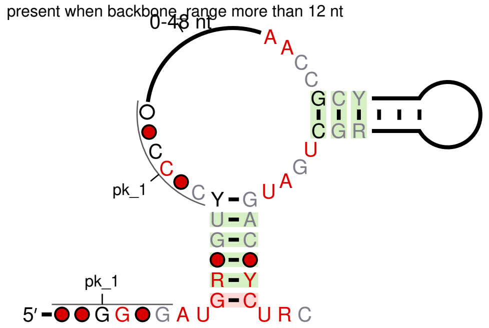

crcB.cons NC_008578.1/805741-806202

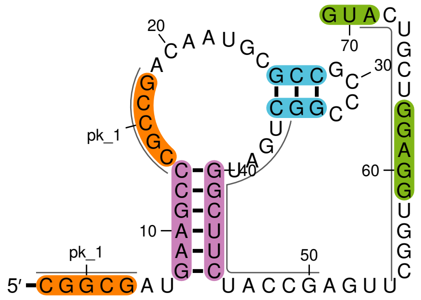

crcB.cons NZ_ABYJ01000311.1/4968-4495

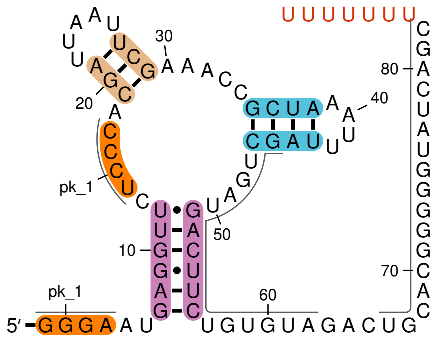

crcB.cons R2R-paper=1

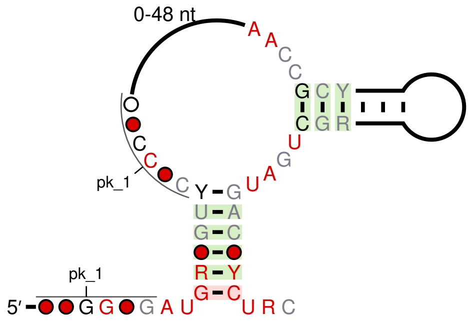

crcB.cons skeleton-with-bp

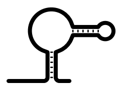

WARNING: R2R is not intended to evaluate evidence for covariation or RNA structure where this is in question. It is not appropriate to use R2R´s covariation markings to declare that there is evidence of structural conservation within an alignment. R2R is a drawing program. As the original paper, Weinberg and Breaker, 2011 wrote:
"This automated R2R annotation [of covariation] does not reflect the extent or confidence of covariation. While such information can be useful, we believe that thorough evaluation of covariation evidence ultimately requires analysis of the full sequence alignment. For example, misleading covariation can result from an incorrect alignment of sequences, or from alignments of sequences that do not function as structured RNAs. Unfortunately, there is no accepted method to assign confidence that entirely eliminates the need to analyze the full alignment."
To disable this warning, run r2r with --disable-usage-warning

THF.cons

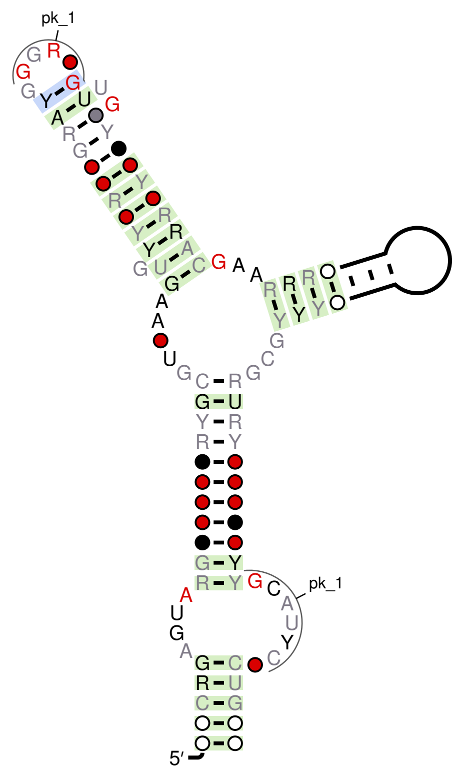

THF.cons 2nt-bulge=1

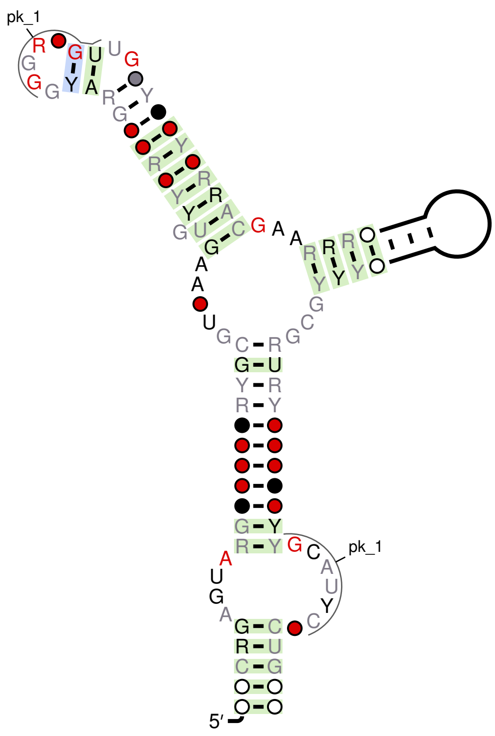

THF.cons 2nt-bulge=2 solver=1

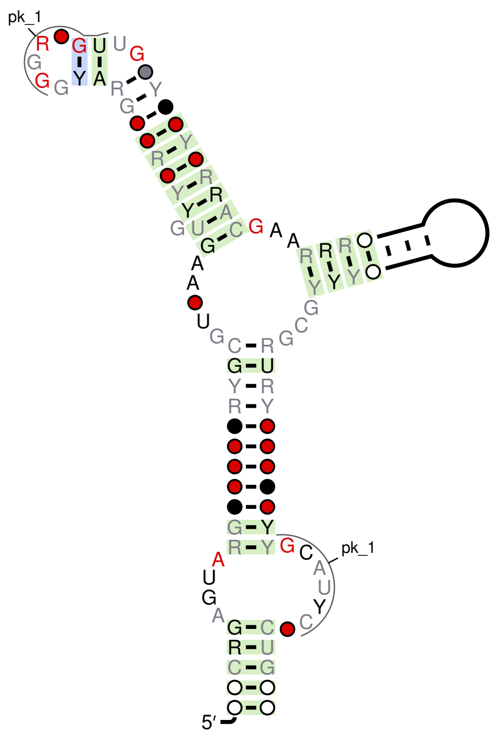

THF.cons pknot=1

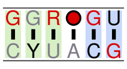

THF.cons short-hairpin=1

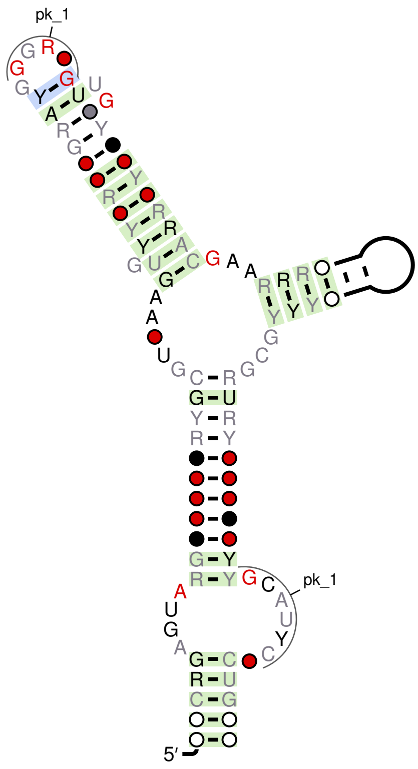

THF.cons skeleton-with-bp

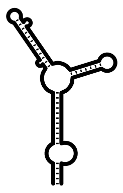

THF.cons skeleton-with-bp 2nt-bulge=1

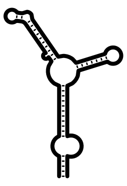

THF.cons solver=1

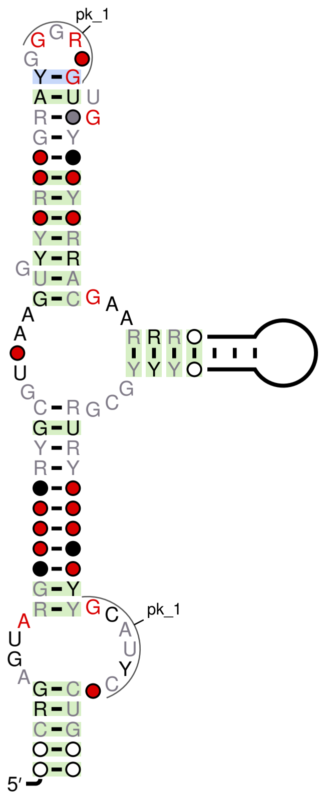

THF.cons solver=2

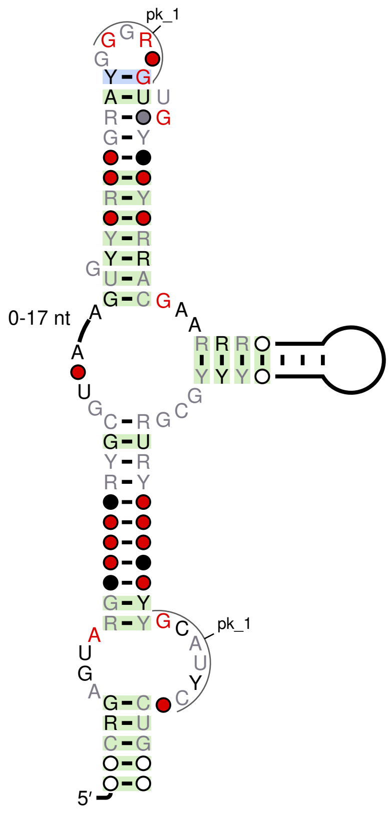

THF.cons solver=2 align-with-circle=1

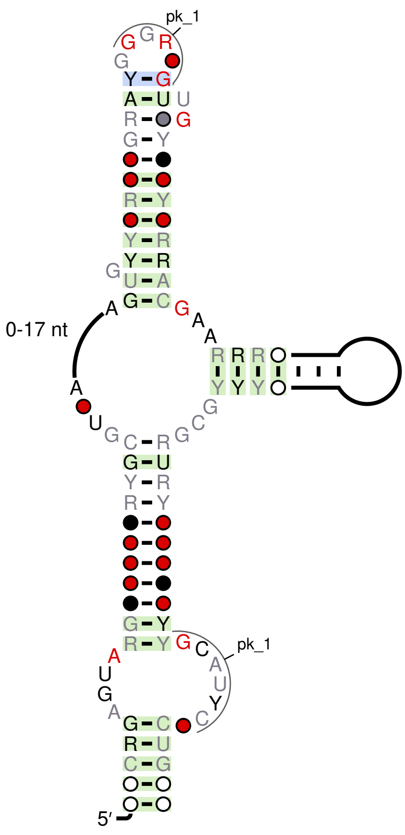

THF.cons solver=3

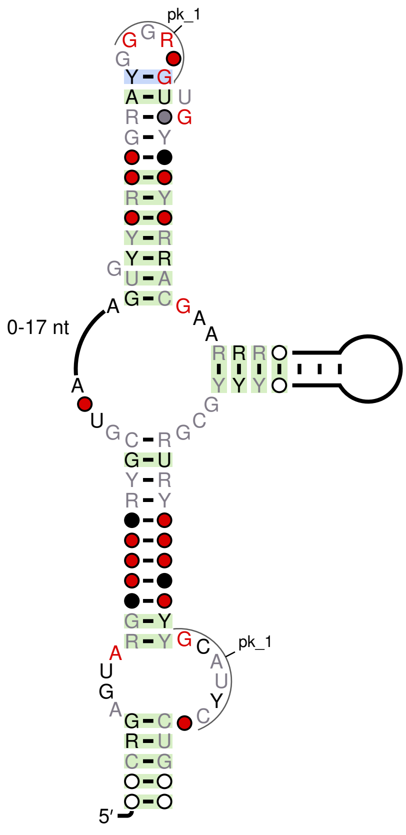

THF.cons solver=3 align-with-circle=1

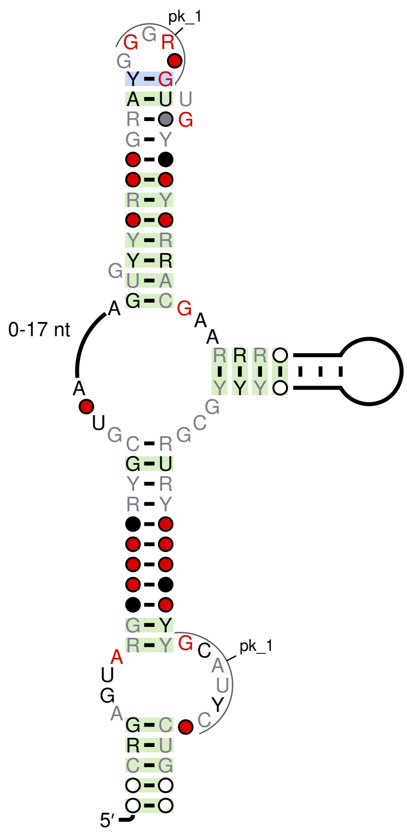

WARNING: R2R is not intended to evaluate evidence for covariation or RNA structure where this is in question. It is not appropriate to use R2R´s covariation markings to declare that there is evidence of structural conservation within an alignment. R2R is a drawing program. As the original paper, Weinberg and Breaker, 2011 wrote: "This automated R2R annotation [of covariation] does not reflect the extent or confidence of covariation. While such information can be useful, we believe that thorough evaluation of covariation evidence ultimately requires analysis of the full sequence alignment. For example, misleading covariation can result from an incorrect alignment of sequences, or from alignments of sequences that do not function as structured RNAs. Unfortunately, there is no accepted method to assign confidence that entirely eliminates the need to analyze the full alignment." To disable this warning, run r2r with
--disable-usage-warning

demo-multistem.cons

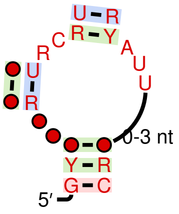

demo-multistem.cons circle=1

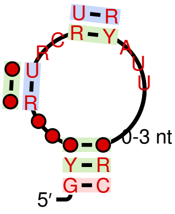

demo-multistem.cons flipstem=1

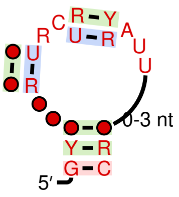

demo-multistem.cons manual=1

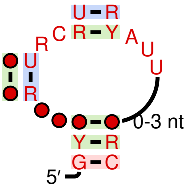

demo-multistem.cons solver1=1

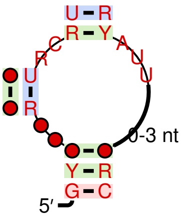

demo-multistem.cons solver2=1

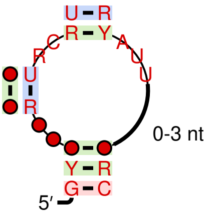

WARNING: R2R is not intended to evaluate evidence for covariation or RNA structure where this is in question. It is not appropriate to use R2R´s covariation markings to declare that there is evidence of structural conservation within an alignment. R2R is a drawing program.
As the original paper, Weinberg and Breaker, 2011 wrote:
"This automated R2R annotation [of covariation] does not reflect the extent or confidence of covariation. While such information can be useful, we believe that thorough evaluation of covariation evidence ultimately requires analysis of the full sequence alignment. For example, misleading covariation can result from an incorrect alignment of sequences, or from alignments of sequences that do not function as structured RNAs. Unfortunately, there is no accepted method to assign confidence that entirely eliminates the need to analyze the full alignment."
To disable this warning, run r2r with --disable-usage-warning

demo-multistem-IMES-1.cons

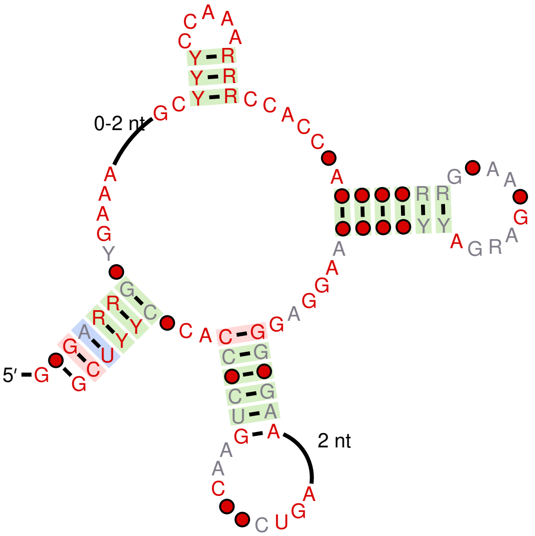

demo-multistem-IMES-1.cons solver=1

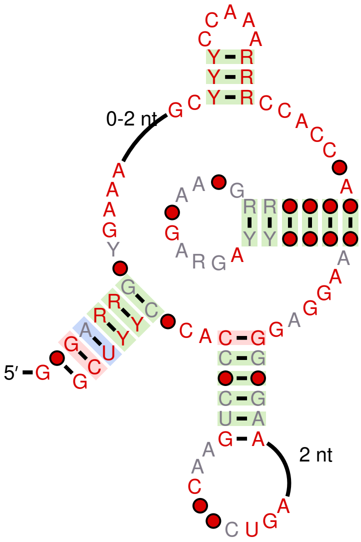

demo-multistem-IMES-1.cons solver=2

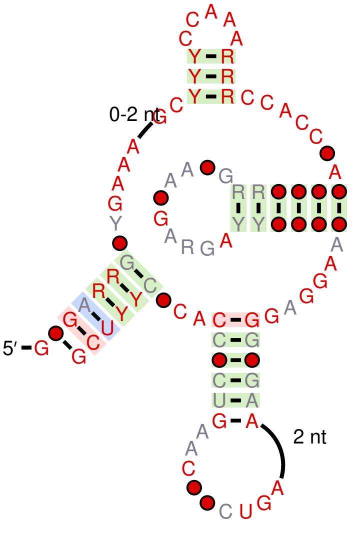

WARNING: R2R is not intended to evaluate evidence for covariation or RNA structure where this is in question. It is not appropriate to use R2R´s covariation markings to declare that there is evidence of structural conservation within an alignment. R2R is a drawing program. As the original paper, Weinberg and Breaker, 2011 wrote:
"This automated R2R annotation [of covariation] does not reflect the extent or confidence of covariation. While such information can be useful, we believe that thorough evaluation of covariation evidence ultimately requires analysis of the full sequence alignment. For example, misleading covariation can result from an incorrect alignment of sequences, or from alignments of sequences that do not function as structured RNAs. Unfortunately, there is no accepted method to assign confidence that entirely eliminates the need to analyze the full alignment."
To disable this warning, run r2r with --disable-usage-warning

c-di-GMP-II-pknot.cons

subfam_weight=1

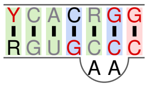

c-di-GMP-II.cons

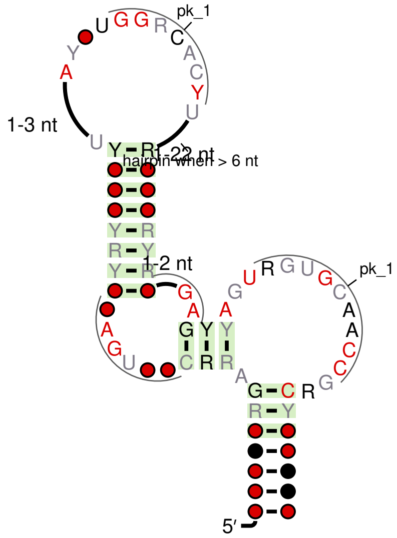

c-di-GMP-II.cons NC_003030.1/648383-648856

Shrinking nucs & bonds using
nucShrinkWithCircleNuc and pairBondScaleWithOneSeq
because circle_nuc or #=GR ... CLEAVAGE was used
To disable this warning message:
#=GF R2R SetDrawingParam nucShrinkWithCircleNuc 1 drawingParams.pairBondScaleWithCircleNuc 1
See note1 in manual for more information.

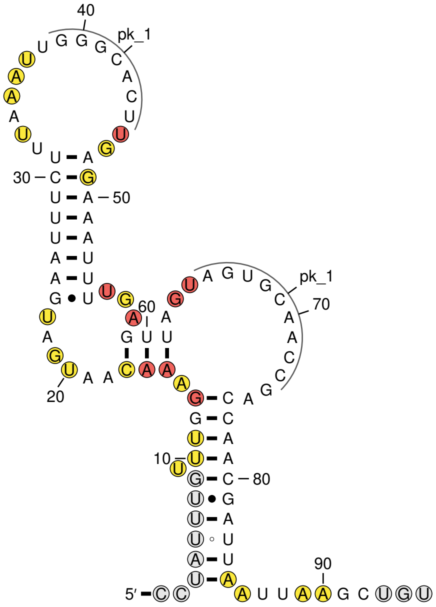

c-di-GMP-II.cons NC_009089.1/3801245-3800770

Shrinking nucs & bonds using
nucShrinkWithCircleNuc and pairBondScaleWithOneSeq
because circle_nuc or #=GR ... CLEAVAGE was used
To disable this warning message:
#=GF R2R SetDrawingParam nucShrinkWithCircleNuc 1 drawingParams.pairBondScaleWithCircleNuc 1
See note1 in manual for more information.

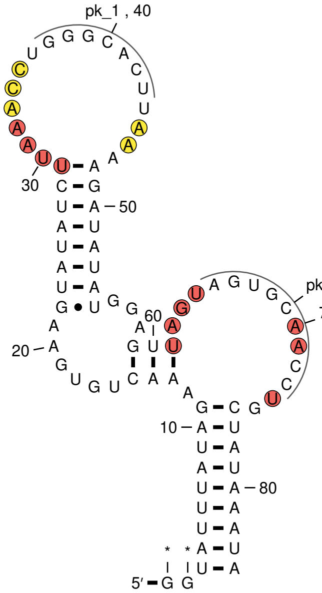

c-di-GMP-II.cons skeleton-with-bp

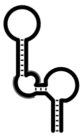

WARNING: R2R is not intended to evaluate evidence for covariation or RNA structure where this is in question. It is not appropriate to use R2R´s covariation markings to declare that there is evidence of structural conservation within an alignment. R2R is a drawing program. As the original paper, Weinberg and Breaker, 2011 wrote:
"This automated R2R annotation [of covariation] does not reflect the extent or confidence of covariation. While such information can be useful, we believe that thorough evaluation of covariation evidence ultimately requires analysis of the full sequence alignment. For example, misleading covariation can result from an incorrect alignment of sequences, or from alignments of sequences that do not function as structured RNAs. Unfortunately, there is no accepted method to assign confidence that entirely eliminates the need to analyze the full alignment."
To disable this warning, run r2r with --disable-usage-warning

c-di-GMP-II-update.cons

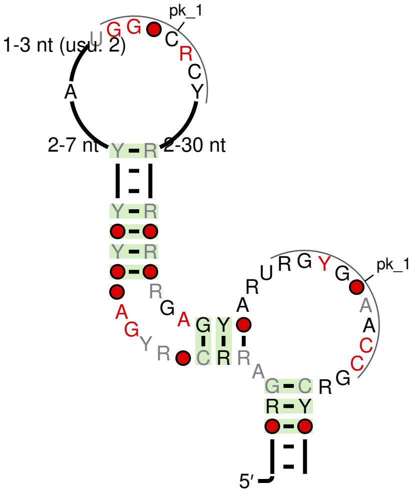

c-di-GMP-II-update.cons pknot=1

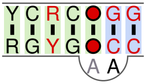

c-di-GMP-II-update.cons skeleton-with-bp

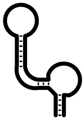

WARNING: R2R is not intended to evaluate evidence for covariation or RNA structure where this is in question. It is not appropriate to use R2R´s covariation markings to declare that there is evidence of structural conservation within an alignment. R2R is a drawing program. As the original paper, Weinberg and Breaker, 2011 wrote:
"This automated R2R annotation [of covariation] does not reflect the extent or confidence of covariation. While such information can be useful, we believe that thorough evaluation of covariation evidence ultimately requires analysis of the full sequence alignment. For example, misleading covariation can result from an incorrect alignment of sequences, or from alignments of sequences that do not function as structured RNAs. Unfortunately, there is no accepted method to assign confidence that entirely eliminates the need to analyze the full alignment."
To disable this warning, run r2r with --disable-usage-warning

solver-formulation1-1.cons

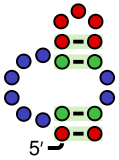

solver-formulation1-1.cons solver=1

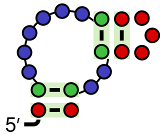

solver-formulation1-1.cons solver=2

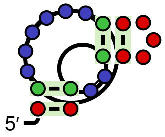

WARNING: R2R is not intended to evaluate evidence for covariation or RNA structure where this is in question. It is not appropriate to use R2R´s covariation markings to declare that there is evidence of structural conservation within an alignment. R2R is a drawing program. As the original paper, Weinberg and Breaker, 2011 wrote:
"This automated R2R annotation [of covariation] does not reflect the extent or confidence of covariation. While such information can be useful, we believe that thorough evaluation of covariation evidence ultimately requires analysis of the full sequence alignment. For example, misleading covariation can result from an incorrect alignment of sequences, or from alignments of sequences that do not function as structured RNAs. Unfortunately, there is no accepted method to assign confidence that entirely eliminates the need to analyze the full alignment."
To disable this warning, run r2r with --disable-usage-warning

uneven-internal-loop seq layout=anyangle

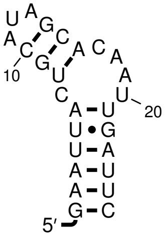

uneven-internal-loop seq layout=broken-left

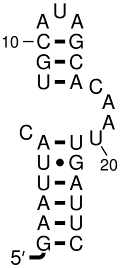

uneven-internal-loop seq layout=default

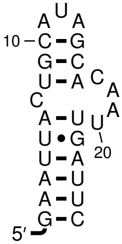

uneven-internal-loop seq layout=left-turn

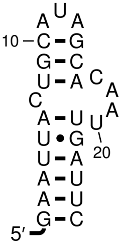

uneven-internal-loop seq layout=left-turn-multistem

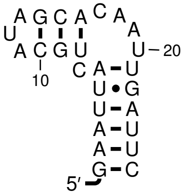

uneven-internal-loop.cons

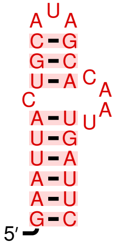

WARNING: R2R is not intended to evaluate evidence for covariation or RNA structure where this is in question. It is not appropriate to use R2R´s covariation markings to declare that there is evidence of structural conservation within an alignment. R2R is a drawing program. As the original paper, Weinberg and Breaker, 2011 wrote:
"This automated R2R annotation [of covariation] does not reflect the extent or confidence of covariation. While such information can be useful, we believe that thorough evaluation of covariation evidence ultimately requires analysis of the full sequence alignment. For example, misleading covariation can result from an incorrect alignment of sequences, or from alignments of sequences that do not function as structured RNAs. Unfortunately, there is no accepted method to assign confidence that entirely eliminates the need to analyze the full alignment."
To disable this warning, run r2r with --disable-usage-warning

TestAngularAlignment.cons

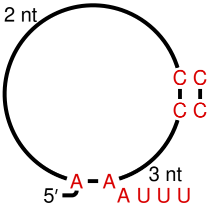

TestAngularAlignment.cons alignangle=-32

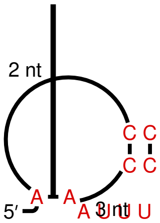

TestAngularAlignment.cons alignangle=-45

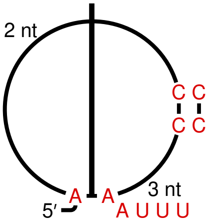

TestAngularAlignment.cons alignangle=45

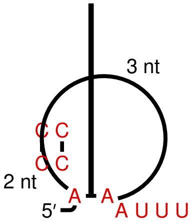

TestAngularAlignment.cons rots0=-15

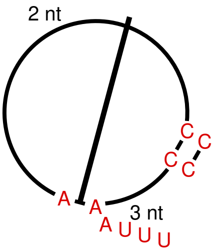

WARNING: R2R is not intended to evaluate evidence for covariation or RNA structure where this is in question. It is not appropriate to use R2R´s covariation markings to declare that there is evidence of structural conservation within an alignment. R2R is a drawing program. As the original paper, Weinberg and Breaker, 2011 wrote:
"This automated R2R annotation [of covariation] does not reflect the extent or confidence of covariation. While such information can be useful, we believe that thorough evaluation of covariation evidence ultimately requires analysis of the full sequence alignment. For example, misleading covariation can result from an incorrect alignment of sequences, or from alignments of sequences that do not function as structured RNAs. Unfortunately, there is no accepted method to assign confidence that entirely eliminates the need to analyze the full alignment."
To disable this warning, run r2r with
--disable-usage-warning

two-glycine Bsu-1

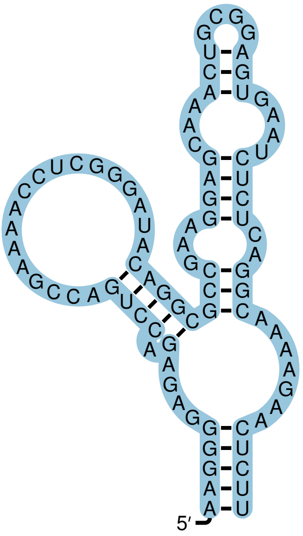

two-glycine Bsu-2

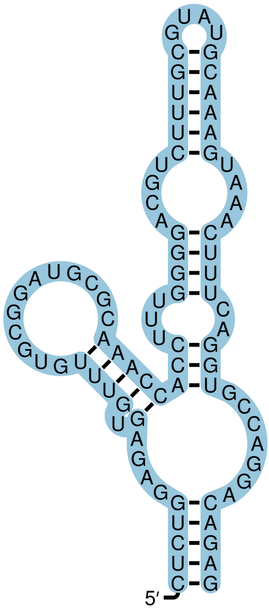

two-glycine.cons

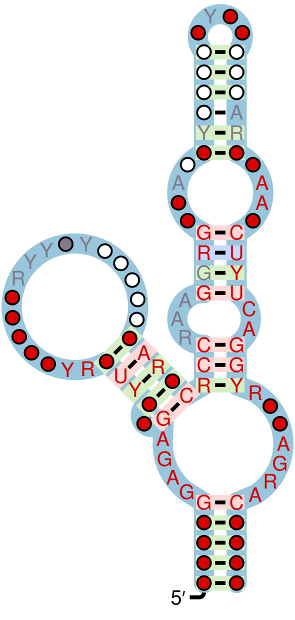

WARNING: R2R is not intended to evaluate evidence for covariation or RNA structure where this is in question. It is not appropriate to use R2R´s covariation markings to declare that there is evidence of structural conservation within an alignment. R2R is a drawing program. As the original paper, Weinberg and Breaker, 2011 wrote:
"This automated R2R annotation [of covariation] does not reflect the extent or confidence of covariation. While such information can be useful, we believe that thorough evaluation of covariation evidence ultimately requires analysis of the full sequence alignment. For example, misleading covariation can result from an incorrect alignment of sequences, or from alignments of sequences that do not function as structured RNAs. Unfortunately, there is no accepted method to assign confidence that entirely eliminates the need to analyze the full alignment."
To disable this warning, run r2r with --disable-usage-warning

solver-formulation2-1.cons

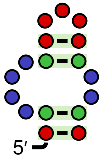

solver-formulation2-1.cons solver=1

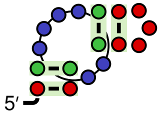

solver-formulation2-1.cons solver=2

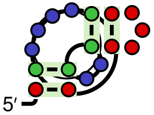

WARNING: R2R is not intended to evaluate evidence for covariation or RNA structure where this is in question. It is not appropriate to use R2R´s covariation markings to declare that there is evidence of structural conservation within an alignment. R2R is a drawing program. As the original paper, Weinberg and Breaker, 2011 wrote:
"This automated R2R annotation [of covariation] does not reflect the extent or confidence of covariation. While such information can be useful, we believe that thorough evaluation of covariation evidence ultimately requires analysis of the full sequence alignment. For example, misleading covariation can result from an incorrect alignment of sequences, or from alignments of sequences that do not function as structured RNAs. Unfortunately, there is no accepted method to assign confidence that entirely eliminates the need to analyze the full alignment."
To disable this warning, run r2r with --disable-usage-warning

demo-multistem-manA.cons

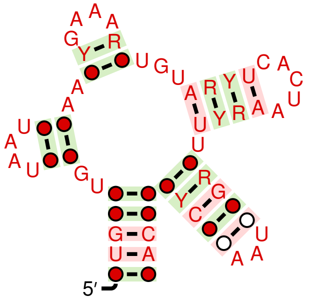

demo-multistem-manA.cons solver=1

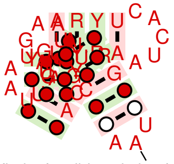

note: R2R cannot find a feasible solution for this problem using the multistem_junction_circular_solver command. However, it can with (1) a user-input optimization starting point, (2) with the try_harder directive or (3) with the multistem_junction_bulgecircley_solver command, as shown in other drawings here.

demo-multistem-manA.cons solver=1explicitinitial

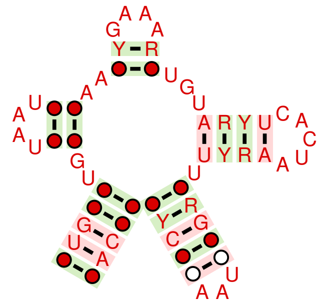

demo-multistem-manA.cons solver=1tryharder

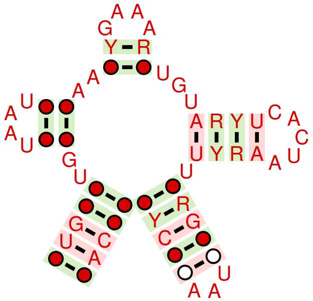

demo-multistem-manA.cons solver=2

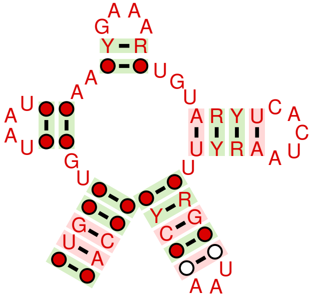

demo-multistem-manA.cons solver=3

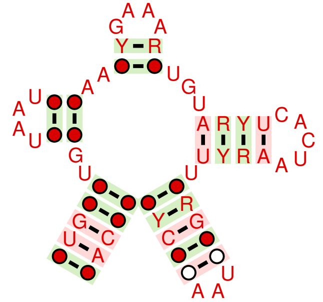

demo-multistem-manA.cons solver=3 annot=1

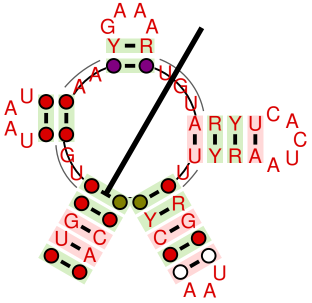

demo-multistem-manA.cons solver=3 annot=1 fewiters=1

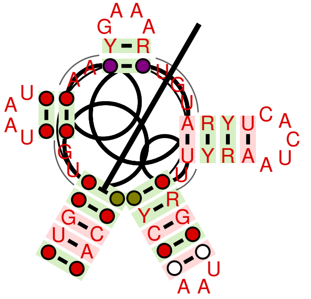

WARNING: R2R is not intended to evaluate evidence for covariation or RNA structure where this is in question. It is not appropriate to use R2R´s covariation markings to declare that there is evidence of structural conservation within an alignment. R2R is a drawing program. As the original paper, Weinberg and Breaker, 2011 wrote:
"This automated R2R annotation [of covariation] does not reflect the extent or confidence of covariation. While such information can be useful, we believe that thorough evaluation of covariation evidence ultimately requires analysis of the full sequence alignment. For example, misleading covariation can result from an incorrect alignment of sequences, or from alignments of sequences that do not function as structured RNAs. Unfortunately, there is no accepted method to assign confidence that entirely eliminates the need to analyze the full alignment."
To disable this warning, run r2r with
--disable-usage-warning

group-II-DV-DVI.cons

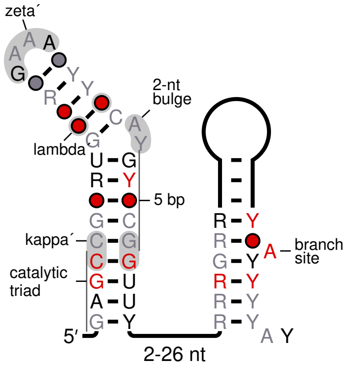

WARNING: R2R is not intended to evaluate evidence for covariation or RNA structure where this is in question. It is not appropriate to use R2R´s covariation markings to declare that there is evidence of structural conservation within an alignment. R2R is a drawing program. As the original paper, Weinberg and Breaker, 2011 wrote:
"This automated R2R annotation [of covariation] does not reflect the extent or confidence of covariation. While such information can be useful, we believe that thorough evaluation of covariation evidence ultimately requires analysis of the full sequence alignment. For example, misleading covariation can result from an incorrect alignment of sequences, or from alignments of sequences that do not function as structured RNAs. Unfortunately, there is no accepted method to assign confidence that entirely eliminates the need to analyze the full alignment."
To disable this warning, run r2r with
--disable-usage-warning

tRNA-var.cons
subfam_weight=0.154412

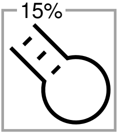

tRNA.cons

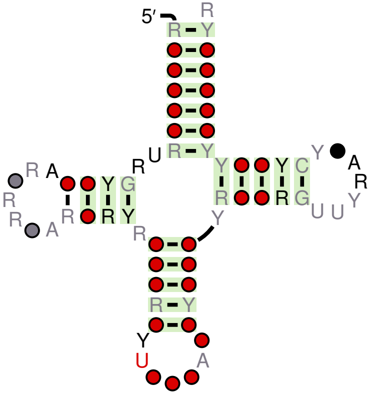

tRNA.cons skeleton-with-bp

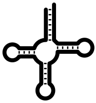

WARNING: R2R is not intended to evaluate evidence for covariation or RNA structure where this is in question. It is not appropriate to use R2R´s covariation markings to declare that there is evidence of structural conservation within an alignment. R2R is a drawing program. As the original paper, Weinberg and Breaker, 2011 wrote:
"This automated R2R annotation [of covariation] does not reflect the extent or confidence of covariation. While such information can be useful, we believe that thorough evaluation of covariation evidence ultimately requires analysis of the full sequence alignment. For example, misleading covariation can result from an incorrect alignment of sequences, or from alignments of sequences that do not function as structured RNAs. Unfortunately, there is no accepted method to assign confidence that entirely eliminates the need to analyze the full alignment."
To disable this warning, run r2r with
--disable-usage-warning

U1.cons

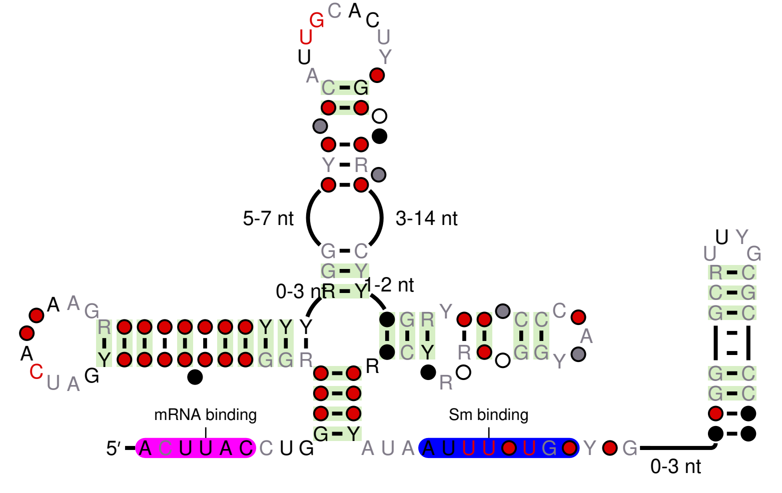

Supplement: Supplement 1 [file media-1.gz › supplemental_material/src/rscape_v2.6.0.tar.gz!/rscape_v2.6.0/lib/R2R/R2R-current/build-package/R2R-1.0.6.cand7-1/demo/solver.pdf]
